# Sustainable methane utilization technology via photocatalytic halogenation with alkali halides

**DOI:** 10.1038/s41467-023-36977-0

**Published:** 2023-03-14

**Authors:** Jun Ma, Can Zhu, Keke Mao, Wenbin Jiang, Jingxiang Low, Delong Duan, Huanxin Ju, Dong Liu, Kun Wang, Yijing Zang, Shuangming Chen, Hui Zhang, Zeming Qi, Ran Long, Zhi Liu, Li Song, Yujie Xiong

**Affiliations:** 1 School of Chemistry and Materials Science, National Synchrotron Radiation Laboratory, School of Nuclear Science and Technology, University of Science and Technology of China, 230026 Hefei, Anhui China; 2 Suzhou Institute for Advanced Research, University of Science and Technology of China, 215123 Suzhou, Jiangsu China; 3 Institute of Energy, Hefei Comprehensive National Science Center, 350 Shushanhu Rd., 230031 Hefei, Anhui China; 4 Department of Chemistry, Fudan University, 2005 Songhu Road, 200438 Shanghai, Yangpu District China; 5 School of Energy and Environment Science, Anhui University of Technology, 243032 Maanshan, Anhui China; 6 PHI China Analytical Laboratory, CoreTech Integrated Limited, 402 Yinfu Road, 211102 Nanjing, Jiangsu China; 7 School of Physical Science and Technology, ShanghaiTech University, 201203 Shanghai, China; 8 State Key Laboratory of Functional Materials for Informatics, Shanghai Institute of Microsystem and Information Technology, Chinese Academy of Sciences, 200050 Shanghai, China; 9 Anhui Engineering Research Center of Carbon Neutrality, College of Chemistry and Materials Science, Key Laboratory of Functional Molecular Solids, Ministry of Education, Anhui Normal University, 241002 Wuhu, Anhui China

**Keywords:** Photocatalysis, Carbon capture and storage, Photocatalysis, Characterization and analytical techniques

## Abstract

Methyl halides are versatile platform molecules, which have been widely adopted as precursors for producing value-added chemicals and fuels. Despite their high importance, the green and economical synthesis of the methyl halides remains challenging. Here we demonstrate sustainable and efficient photocatalytic methane halogenation for methyl halide production over copper-doped titania using alkali halides as a widely available and noncorrosive halogenation agent. This approach affords a methyl halide production rate of up to 0.61 mmol h^−1^ m^−2^ for chloromethane or 1.08 mmol h^−1^ m^−2^ for bromomethane with a stability of 28 h, which are further proven transformable to methanol and pharmaceutical intermediates. Furthermore, we demonstrate that such a reaction can also operate solely using seawater and methane as resources, showing its high practicability as general technology for offshore methane exploitation. This work opens an avenue for the sustainable utilization of methane from various resources and toward designated applications.

## Abstract (web summary)

A sustainable photocatalytic methane halogenation strategy is developed for methyl halide production using low-cost alkali halides over Cu-doped TiO2 nanostructures. Copper sites ultimately stabilize *CH3 to promote reaction with Cl− to form CH3Cl.

## Introduction

Methyl halides, such as chloromethane (CH_3_Cl) and bromomethane (CH_3_Br), represent versatile platform molecules for producing various value-added chemicals and fuels (e.g., light olefins, pharmaceutical intermediates and organoboron agents)^1–4^. Industrially, the production of methyl halides primarily involves methanol as feedstock, which is also precious fuel and chemical, along with complicated process and intensive energy input, inducing high capital cost (Supplementary Fig. 1a)^5,6^. Against this background, methane (CH_4_), whose reserves have been recently bloomed due to the exploration of methane hydrate, shale gas and coalbed methane, has emerged as a potential substitution of methanol as carbon feedstock for methyl halide production^7–12^. Specifically, Olah and coworkers pioneeringly reported the possibility of catalytic methane halogenation for methyl halide production, paving a way for cheap and effective production of methyl halides^13^.

Since then, a series of important advances have been achieved in methane halogenation, pushing forward its development toward practical applications^1,3,14–17^. For example, Zhang and coworkers employed FePO_4_/SiO_2_ for oxybromination of methane at 570 °C using HBr as halogen agent, which achieved a methane conversion of 50% and a selectivity of 48% for CH_3_Br production^18^. Photochemical technology was also employed in methane halogenation using halide under UV irradiation (Supplementary Fig. 1b)^19–21^. Notwithstanding its rapid advancement, there are still critical limitations (e.g., corrosive feed gas, high working temperature and unsatisfactory selectivity) which defer the methane halogenation toward substituting the conventional industrial methyl halide production^22,23^. The decisive limitation is the involvement of dangerous feed gas (e.g., Cl_2_, HCl, and HBr) in the reaction, which can lead to potential hazards and corrosion of catalysts and reactors. Therefore, it is an urgent task to develop safe and sustainable approaches for methane halogenation using widely available halogen sources.

**Fig. 1:**
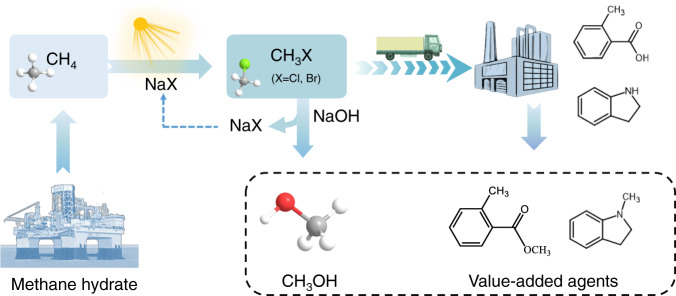
Schematic illustration for the proposed concept. The photocatalytic methane halogenation using alkali halide as a halogenation agent at room temperature, and the successive transformations into fuels and chemicals.

Alkali halides, especially sodium halide, represent a class of green and cheap halogen sources, yet their inertness greatly hinder their utilization in methane halogenation. Although a previous report suggested that the methane chlorination with metal chlorides may be achieved by UV-responsive photocatalyst, the reported case only exhibited low product selectivity and catalyst durability, and as such, the products can hardly be continuously converted into value-added chemicals and fuels^18^. Here, we present a simple yet effective photocatalytic route for activating alkali halides, and utilize alkali halides as halogen source for methane halogenation. Specifically, the Cu-doped porous TiO_2_ photocatalyst (Cu–TiO_2_) is employed to drive the methane halogenation under moderate conditions (i.e., room temperature and ambient pressure) (Fig. 1 and Supplementary Fig. 1c). We adopt the photocatalytic methane chlorination as a proof-of-concept example using sodium chloride as halogen agent, achieving a methyl chloride production rate of 0.61 mmol h^−1^ m^−2^ with a selectivity of 83.7% upon light irradiation. In situ spectroscopic characterizations and theoretical simulations manifest that the methane molecule can be first dissociated into methyl intermediate on the surface of the photocatalyst. In addition, evidenced by ambient pressure X-ray photoelectron spectroscopy (AP-XPS), the halogen ions (i.e., chloride in this case) from alkali halides can be oxidized and activated by photogenerated holes from the photocatalyst to form active chloride species. As such, the produced active chloride species can react with the methyl intermediates to produce methyl chloride. This approach enables the direct use of seawater and methane feedstocks for methyl chloride production, largely extending its practical applications such as offshore methane exploitation. Moreover, we construct reaction systems for production of methanol and pharmaceutical intermediates (i.e., methyl o-toluate and 1-methylindole) via a methyl halide mediated pathway. This work provides an approach for selective methane halogenation under mild conditions using green and cheap alkali halide as halogen source, shedding significant light on sustainable methyl halide production.

## Results

### Catalyst synthesis and characterization

In this work, the Cu, which has been proven to play a critical role as active sites for methane activation on particulate methane monooxygenase in nature, is selected to dope TiO_2_ photocatalysts for methane halogenation^15,22^. The Cu-doped porous TiO_2_ nanostructures (denoted as *X*%Cu–TiO_2_, where *X* represents the molar percentage of Cu atoms in precursors) are synthesized in gram-scale via a simple sol–gel process (Supplementary Fig. 2a). Transmission electron microscopy (TEM) images (Supplementary Figs. 2b, c, 3) reveal the porous morphology of as-synthesized samples. As determined by nitrogen adsorption–desorption isotherms, all the prepared samples exhibit large specific surface area with a pore size distribution of ca. 10 nm (Supplementary Fig. 4 and Table 1), endowing enormous active sites for photocatalytic reaction.

Energy-dispersive X-ray spectroscopy (EDS) mapping suggests the presence of Ti, O and Cu elements (Supplementary Fig. 2d), affirming the composition of the prepared samples. According to powder X-ray diffraction (XRD) characterization (Supplementary Fig. 2e), the crystal phase of TiO_2_ maintains unchanged after introduction of Cu elements. In addition, as revealed by UV–vis–NIR diffuse reflectance spectroscopy (Supplementary Fig. 5), the light absorption edges of the *X*%Cu–TiO_2_ red shift with the increasing Cu concentration due to the presence of oxygen vacancies (*V*_O_) (Supplementary Fig. 6) and 2E_*g*_ → 2T_2*g*_ transitions from O to Cu atoms^24^. Moreover, the enhanced absorptions of the samples in the visible–near-infrared regions are attributed to the d–d transitions of the Cu dopants^24^. Synchrotron-radiation photoemission spectroscopy (SRPES) indicates that the valence-band maxima (VBM) of 0%Cu–TiO_2_ and 2%Cu–TiO_2_ are 3.56 and 3.46 eV below the Fermi level (*E*_F_), respectively (Supplementary Fig. 7). Combined with the determined band gaps from light absorption spectra (Supplementary Fig. 8), the band structure changes of TiO_2_ before and after Cu-doping can be estimated, showing that the Cu-doping can slightly shift both the VBM and conduction band minimum (CBM) of TiO_2_ (Supplementary Fig. 9). Furthermore, X-ray photoelectron spectroscopy (XPS), X-ray absorption spectroscopy (XAS) and X-ray absorption fine structure (XAFS) spectroscopy confirm that the Cu is doped into the TiO_2_ matrix as a single-site substitution and exhibits a +2 oxidation state in 2%Cu–TiO_2_ (Supplementary Figs. 10–12). Furthermore, the Fourier-transformed (FT) Ti K-edge extended XAFS (EXAFS) spectra of the 0%Cu–TiO_2_ and 2%Cu–TiO_2_ demonstrate that the Ti–O coordination numbers of TiO_2_ decrease from 3.98 to 3.08 after Cu doping (Supplementary Fig. 2f, g and Supplementary Tables 2, 3), suggesting the existence of oxygen vacancies. This result collaborates that the Cu-doping can induce the formation of oxygen vacancies on the TiO_2_.

### Photocatalytic performance

**Fig. 2:**
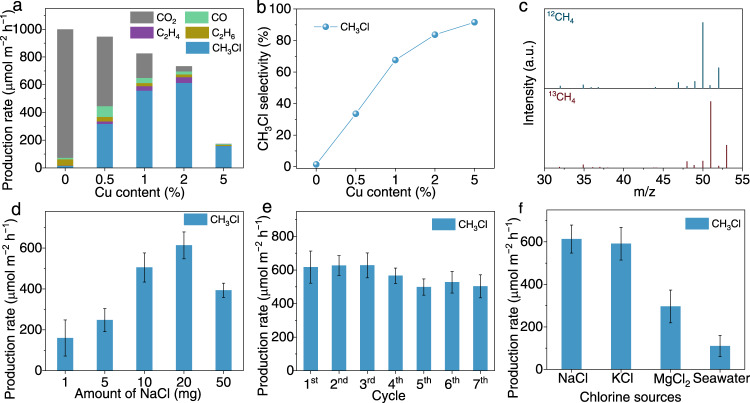
Photocatalytic halogenation of CH_4_ under different conditions. **a**, **b** Photocatalytic methane halogenation efficiency (**a**) and selectivity (**b**) of *X*%Cu–TiO_2_ toward CH_3_Cl production. **c** Mass spectra of ^12^CH_3_Cl (*m/z* = 50) and ^13^CH_3_Cl (*m/z* = 51) produced over 2%Cu–TiO_2_ via photocatalytic methane halogenation. **d** Production rates of CH_3_Cl via photocatalytic methane halogenation over 2%Cu–TiO_2_ with different amounts of NaCl. **e** The cyclic test of photocatalytic CH_3_Cl production over 2%Cu–TiO_2_. **f** Photocatalytic production rates of CH_3_Cl via methane halogenation over 2%Cu–TiO_2_ using various chlorine sources. The light intensity is 600 mW cm^−2^ for all the measurements here. Error bars indicate standard deviations.

After recognizing the physicochemical properties of prepared samples, we are now in a position to evaluate their applicability in photocatalytic methane halogenation. The photocatalytic experiments are carried out through a gas-solid phase reaction using sodium halide (e.g., NaCl and NaBr) as halogen agent at ambient conditions (Supplementary Figs. 13, 14). The test is first performed by varying doping parameters to screen optimal photocatalysts under the light illumination of 600 mW cm^−2^. As shown in Fig. 2a and Supplementary Fig. 15, the 0%Cu–TiO_2_ shows negligible CH_3_Cl production, while the CH_3_Cl production rate gradually increases with the increase of Cu content from 0.5 to 2%, suggesting the important role of Cu dopants in triggering methane halogenation. Specifically, the 2%Cu–TiO_2_ achieves a methyl chloride production rate of 0.61 mmol h^−1^ m^−2^ with a selectivity of 83.7%. Yet, the overdose of Cu dopants (5%Cu–TiO_2_) can lead to photogenerated charge recombination^25^, resulting in decrease in CH_3_Cl production rate. The selectivities toward the CH_3_Cl production of the prepared samples shown in Fig. 2b imply the strong correlation of the CH_3_Cl production with Cu contents, further confirming the critical role of Cu in such a reaction (Supplementary Fig. 16). The performance of photocatalytic methane chlorination over CuO-loaded TiO_2_ nanostructures is far lower than that of the Cu–TiO_2_ (Supplementary Fig. 17), indicating the significant role of Cu doping in methane and halide activation that induces oxygen vacancies with localized charge trapping centers on TiO_2_. Moreover, the apparent quantum efficiency (AQE) over 2%Cu–TiO_2_ is measured to be 5.4% at 254 nm and 0.3% at 365 nm monochromatic light irradiation. To clarify the accuracy of our obtained results, we conduct the isotope labeling test using ^12^CH_4_ or ^13^CH_4_ as the feed gas to perform photocatalytic halogenation of CH_4_ over 2%Cu–TiO_2_ (Fig. 2c). The location of the strongest peak changes from *m/z* = 50 (using ^12^CH_4_ as fed gas) to *m/z* = 51 (using ^13^CH_4_ as fed gas), verifying that the carbon source of generated CH_3_Cl indeed originates from CH_4_.

Upon identifying the optimal photocatalyst, we evaluate the influence of experimental parameters on the photocatalytic methane halogenation performance of the optimized sample (i.e., 2%Cu–TiO_2_). As revealed in Fig. 2d, the 20 mg is the optimal loading content of the NaCl in the system. The overloading of NaCl (i.e., 50 mg) can lead to the decrease in the photocatalytic methane halogenation performance due to the occupied surface active sites on the photocatalyst by the NaCl. In addition, it is unveiled that the high intensity of light irradiation is beneficial for optimizing the photocatalytic methane halogenation, elevating the methyl chloride production rate up to 0.69 mmol h^−1^ m^−2^ at 800 mW cm^−2^ (Supplementary Fig. 18a). It is worth mentioning that the Cu–TiO_2_ catalyst can drive photocatalytic halogenation of methane under visible light irradiation (Supplementary Fig. 18b). Moreover, we have performed a time-dependent measurement with the continued light irradiation up to 14 h. As shown in Supplementary Fig. 19, the CH_3_Cl production gradually increases and then becomes steady with the evolution of reaction time. In the meantime, the amount of H_2_, generated during photocatalytic methane chlorination, is nonstoichiometric to the amount of methyl chloride (Supplementary Fig. 20), which results from probable consumption of lattice oxygen in TiO_2_ during photocatalytic CH_4_ chlorination. The recovery of consumed lattice oxygen can be easily conducted in the air to regenerate the pristine structure of Cu–TiO_2_. Interestingly, the production rate of CH_3_Cl obviously decreases to 0.44 mmol h^−1^ m^−2^ along with negligible H_2_ production (Supplementary Fig. 21a) when the photocatalytic methane halogenation is performed in the absence of H_2_O. In addition, the CH_3_Cl production gradually becomes steady at about 8 h (Supplementary Fig. 21b), indicating the significant role of H_2_O in photocatalytic methane halogenation reaction over Cu–TiO_2_ catalyst.

The practical applicability of the Cu–TiO_2_ for photocatalytic methane halogenation is also evaluated by the recycling test, demonstrating the high photocatalytic stability of the system. The photocatalytic performance only shows a minor decrease after four cycles of reaction, which may result from slight loss of Cu element during cyclic test, and then becomes stable over the successive three cycles (Fig. 2e, Supplementary Fig. 22, Supplementary Tables 4, 5). Furthermore, we employ the KCl, MgCl_2_ or seawater (containing water and rich NaCl), as chlorine sources. Surprisingly, such combinations of reactants can also allow the methane halogenation to proceed over Cu–TiO_2_ (Fig. 2f), firmly implying the sustainability and low cost of this approach for photocatalytic methane halogenation. To further confirm the universality of the photocatalytic methane halogenation, several sodium halides (i.e., NaF, NaCl and NaBr) are also applied for methane halogenation. As shown in Supplementary Fig. 23, the performance of methane halogenation using NaBr shows the highest methyl halide production rate (1.08 mmol h^−1^ m^−2^), while the performance using NaF shows the lowest. These results suggest the universality of the photocatalytic methane halogenation and the strong correlation between halogenation efficiency and electronegativity of halogen. It is worth noting that, as compared with thermocatalytic methane halogenation using halogens or hydrogen halides, photocatalytic methane halogenation using alkali halides exhibits lower conversion and productivity (Supplementary Table 6). However, photocatalytic methane halogenation process can still find its specific application given the use of solar energy as power input and alkali halides as halogen source. For instance, offshore methane exploitation usually does not afford the power input for thermocatalytic conditions.

### In situ characterization toward mechanism

**Fig. 3:**
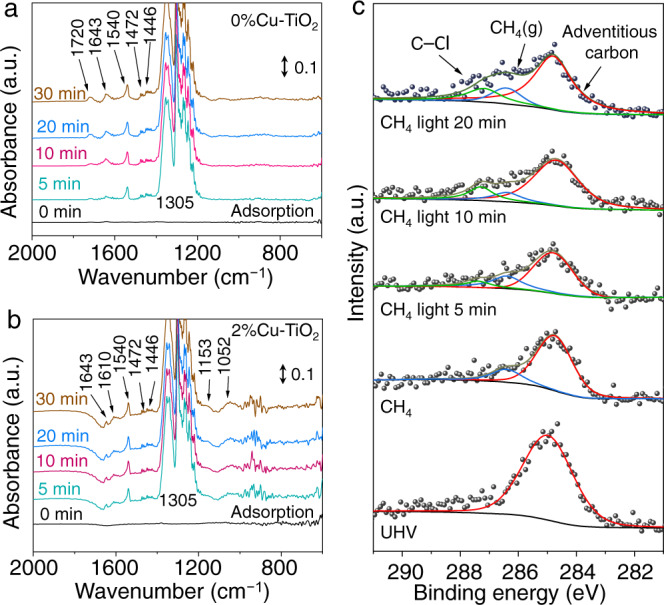
In situ characterization of photocatalytic CH_4_ halogenation. **a**, **b** In situ DRIFTS spectra for photocatalytic methane halogenation over 0%Cu–TiO_2_ (**a**) and 2%Cu–TiO_2_ (**b**) using NaCl as halogenation agent under dark condition. **c** The C 1*s* region of AP-XPS spectra for 2%Cu–TiO_2_ under different conditions.

To look into the reaction mechanism, we perform synchrotron-radiation-based in situ diffuse reflectance infrared Fourier-transform spectroscopy (DRIFTS) to monitor the reaction species evolution on the catalyst surface during the photocatalytic methane halogenation. Under dark condition (i.e., reactant adsorption), both 0%Cu–TiO_2_ and 2%Cu–TiO_2_ exhibit obvious peaks at ca. 1305, 1540 and 1446/1472 cm^−1^ (Fig. 3a, b), assigned to the C–H deformation vibration of CH_4_, C–H symmetric deformation vibrational mode of CH_4_, and CH_3_/CH_2_ deformation vibrational modes, respectively^26–29^. Apart from these similarities, obvious differences can be observed on the DRIFTS spectra of 0%Cu–TiO_2_ and 2%Cu–TiO_2_. For 0%Cu–TiO_2_, two peaks at ca. 1643 and 1720 cm^−1^ assigned to C = O stretching vibration can be observed, demonstrating the formation of carboxylate species on the 0%Cu–TiO_2_ surface^26,27,30^. These carboxylate species formed during reactant adsorption can be easily transformed into CO_2_ during the photocatalytic methane halogenation reaction, collaborating the above-obtained photocatalytic methane halogenation results of 0%Cu–TiO_2_, which is dominated with high CO_2_ production selectivity^28,31,32^. In contrast, three additional peaks can be observed on the DRIFTS spectra of 2%Cu–TiO_2_. At 1045 cm^−1^, a peak attributed to hydroxyl groups (–OH) stretching can be observed, indicating that the Cu can promote the transformation of adsorbed CH_4_ into methyl species (–CH_3_) and –OH, and suppress the formation of C = O and C–O species^33,34^. At 1153 and 1610 cm^−1^, two peaks assigned to methoxy species (–OCH_3_) are identified, implying that a small fraction of adsorbed methane is dissociated into –OCH_3_^30,35^. When turning on the light, a peak at 715 cm^−1^, assigned to C–Cl bond, can be observed and gradually grows over 2%Cu–TiO_2_ upon light irradiation, further suggesting the generation of CH_3_Cl from photocatalytic methane halogenation^36,37^, while no significant changes can be observed for other species on the samples (Supplementary Fig. 24). These results manifest the essential role of Cu in dissociating methane molecules into hydroxyl and methyl species during the reactant adsorption on the catalyst surface.

As mentioned in the previous section, the Cu doping can cause the formation of oxygen vacancies on the Cu–TiO_2_. To this end, the in situ electron paramagnetic resonance (EPR) spectroscopy measurements are performed to reveal the evolution of oxygen vacancies during the reaction. The EPR spectra collected under dark and light irradiation conditions in CH_4_ atmosphere for 0%Cu–TiO_2_ and 2%Cu–TiO_2_ are shown in Supplementary Fig. 25. Under dark condition, both 0%Cu–TiO_2_ and 2%Cu–TiO_2_ exhibit obvious signals at *g* = 2.00 assigned to single electron-trapped surface defects (V_O_^+^ or O^−^)^38,39^, while 2%Cu–TiO_2_ shows an additional broad characteristic Cu^2+^ signal at *g* = 2.15^25,40^. Upon light irradiation, the V_O_^+^ or O^−^ signal shows no detectable change for *X*%Cu–TiO_2_. Meanwhile, the signal of Cu^2+^ for 2%Cu–TiO_2_ distinctly decreases, revealing the reduction of Cu^2+^ into EPR-silent Cu^+^ by photogenerated electrons^41^. Such a result suggests that the Cu dopant can accept the photogenerated electrons from the TiO_2_ during the photocatalytic reaction, significantly enhancing the separation of photogenerated carriers.

To gain further insight into the photocatalytic methane halogenation pathway, the AP-XPS is performed to investigate the evolution of methane molecule and chlorine on the surface of 2%Cu–TiO_2_ during the reaction (Supplementary Fig. 26)^42^. As shown in Fig. 3c, the C 1*s* AP-XPS spectrum collected in ultra-high vacuum (UHV) exhibits a peak at 284.8 eV, assigned to the adventitious carbon on the catalyst. Upon flowing CH_4_ gas and H_2_O vapor into the system, a new peak at 286.6 eV attributed to gaseous CH_4_ appears. Upon light irradiation, a C 1*s* peak at 287.2 eV appears and gradually evolves, suggesting the stable formation of C–Cl bond^43–45^. For the Cl 2*p* AP-XPS spectrum, a typical NaCl peak can be found during the adsorption stage. Upon light irradiation, the Cl 2*p* spectrum becomes broader and slightly shifts toward higher binding energy (Supplementary Fig. 27), suggesting that the Cl ions of NaCl are oxidized by the photogenerated holes from 2%Cu–TiO_2_. Such oxidized Cl species can subsequently couple with methyl groups (–CH_3_), derived from dissociated methane, to form methyl chloride (CH_3_Cl) under light irradiation, which is also confirmed by the slight shift of Cl 2*p* XPS peak toward higher binding energy as compared with NaCl (Supplementary Fig. 27).

### Mechanism investigation

**Fig. 4:**
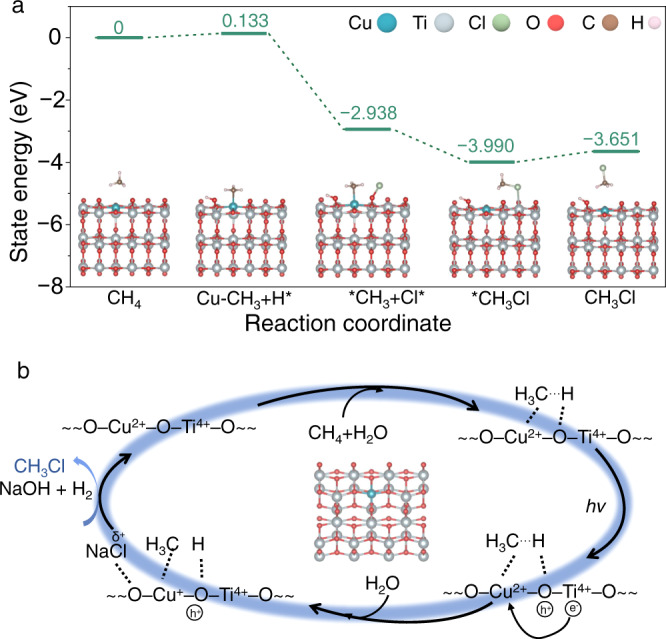
Proposed mechanism for photocatalytic CH_4_ halogenation. **a** The DFT calculations for the production of CH_3_Cl in photocatalytic methane halogenation on Cu-doped TiO_2_. The insets show the optimized structures for each step. **b** The schematic illustration of the proposed mechanism for photocatalytic methane halogenation over Cu-doped TiO_2_.

To further elucidate the reaction mechanism, the density functional theory (DFT) calculations are carried out to depict the energy profile of photocatalytic methane halogenation. We first calculate the dissociation energy of methane molecule on different photocatalysts. For the pristine TiO_2_, although CH_4_ molecule can be dissociated into *CH_3_ with an electron energy change of 0.132 eV, the formed *CH_3_ intermediates can hardly be stabilized (Supplementary Fig. 28). In contrast, the formed *CH_3_ intermediates can be stabilized on the Cu–TiO_2_ with the binding of their C and H atoms to Cu and O atoms of Cu–TiO_2_, respectively (Fig. 4a). These results distinctly confirm that the role of Cu as intermediate stabilization sites during the dissociation of methane molecules. Based on the AP-XPS characterization, the Cl ions of NaCl are oxidized into *Cl intermediates by the photogenerated holes upon light irradiation. As such, the DFT calculations are performed to examine the activation of Cl ions on the surface of Cu–TiO_2_ catalyst under light irradiation. As shown in the Supplementary Fig. 29, the Cl ions from NaCl cluster on the surface of Cu–TiO_2_ tend to lose the electrons and exhibit an oxidized state. Subsequently, these *Cl intermediates can couple with the stabilized *CH_3_, forming *CH_3_Cl with its C atom binding to Cu site with an electron energy change of −1.052 eV. Finally, the CH_3_Cl molecules can be facilely desorbed from the surface of photocatalysts with a small energy change of 0.339 eV. Notably, the presence of H_2_O can provide hydrogen source for H_2_ production and oxygen source for regeneration of lattice oxygen. In detail, the H_2_O can be first adsorbed on the oxygen vacancy, derived from the consumption of lattice oxygen during the generation of CH_3_Cl and NaOH over Cu–TiO_2_, which then is dissociated into OH* and (O)H* species neighboring the oxygen vacancy with an electron energy change of −1.498 eV (Supplementary Fig. 30). Upon irradiation, the H_2_ can be formed and desorbed from the Cu–TiO_2_ surface^46^, while the oxygen from water will be filled into the lattice to regenerate the pristine structure of Cu–TiO_2_. Given that the methoxy species are also resolved from the in situ DRIFTS characterization using Cu–TiO_2_, the reaction route for photocatalytic methane halogenation based on methoxy species is also calculated. As shown in Supplementary Fig. 31, methane molecule can be easily dissociated into methoxy species (*O–CH_3_) on the surface of Cu–TiO_2_, which is exergonic with an electron energy change of −2.280 eV. However, the strong binding of the methoxy species (−5.059 eV) on the Cu–TiO_2_ surface restricts them to proceed with the following transformations.

Taken together, in situ characterizations and DFT calculations indicate that the Cu dopants play a critical role for guiding the formation of the CH_3_Cl during the photocatalytic methane halogenation. Specifically, during the reactant adsorption, the CH_4_ molecules are first dissociated on the surface of Cu–TiO_2_, forming methyl and hydroxyl species (Fig. 4b). These intermediate species can subsequently be stabilized by the Cu and TiO_2_. Upon light irradiation, the photogenerated holes on the Cu–TiO_2_ can oxidize the NaCl to form active halogen species, which can couple with methyl to form methyl chloride; simultaneously, the photogenerated electrons transferred from TiO_2_ to Cu can be utilized for reducing the H to produce H_2_.

## Discussion

Methyl halide, a highly versatile platform molecule, can be facilely transformed into value-added chemicals (e.g., fuels, pharmaceutical intermediates and organoboron agents) under relatively mild conditions (Supplementary Table 7)^14,23^. To show the high feasibility of our strategy, we construct a cycling reaction system for photocatalytic methane conversion into methanol via a methyl chloride mediated pathway. As shown in Supplementary Fig. 32, methyl chloride is first obtained through photocatalytic methane halogenation. Subsequently, methyl chloride can be directly mixed with the NaOH aqueous solution, forming methanol. As an outcome, methanol can be produced with a production rate of 0.51 mmol h^−1^ m^−2^ via the constructed methane conversion system at 80 °C (Supplementary Fig. 33). It should be noted here that the H_2_ and NaCl are the only two byproducts of the overall reaction. The H_2_ can be used as the fuels, while the NaCl can be recycled into the reaction system for sustaining the overall reaction (Supplementary Fig. 32). Given that no any contaminated feedstock or byproducts can be found in such a reaction system, it is a green and sustainable methane conversion approach.

Apart from fuel production, methyl halide can be also applied for synthesizing high-value pharmaceutical intermediates at mild conditions. To further show the multi-purpose features of methyl halide in chemical manufacturing, we demonstrate the synthesis of methyl o-toluate and 1-methylindole (Entry 2 and 3 in Supplementary Table 7) which are essential intermediates for anti-cancer drugs, using methyl halide as precursors. As shown in Supplementary Figs. 34–37, high selectivity toward methyl o-toluate and 1-methylindole can be obtained under mild conditions (0 °C– room temperature) in N,N-dimethylformamide solution over CH_3_Br.

In summary, we present a selective and sustainable photocatalytic methane halogenation strategy for methyl halide production using cost-effective and noncorrosive NaCl over Cu-doped porous TiO_2_ nanostructures. In details, a methyl chloride production rate of 0.61 mmol h^−1^ m^−2^ and a methyl bromide production rate of 1.08 mmol h^−1^ m^−2^ are achieved using optimized Cu–TiO_2_ at room temperature and ambient pressure. On the basis of in situ characterization techniques and theoretical calculation, it is confirmed that the Cu plays a critical role in such a reaction, where it can be utilized to stabilize the *CH_3_ for its coupling with Cl^−^ to form the targeted CH_3_Cl. This work presents a fresh perspective for photocatalytic activation of alkali halides toward methane halogenation under mild conditions, and paves the way for efficient methane conversion into value-added chemicals as well as offshore methane exploitation through a highly sustainable approach.

## Methods

### Material synthesis

The Cu-doped porous TiO_2_ nanostructures (*X*%Cu–TiO_2_) were synthesized through a sol-gel method. In a typical synthesis, 2.4 g F127 was dissolved in 30 mL ethanol under stirring. Then 3.4 mL Ti(OBu)_4_, a specific amount of Cu(NO_3_)_2_·3H_2_O, 2.3 mL acetic acid and 2.0 mL hydrochloric acid were added into the F127 solution. The mixture was stirred vigorously for 2 h and transferred into a petri dish (diameter 125 mm), and heated on a hotplate at 40 °C for 12 h. After evaporation of ethanol, a transparent gel was formed, and was transferred into a 65 °C oven for additional 24 h aging. Finally, the gel was calcinated at 450 °C in the air for 5 h with a ramp rate of 2 °C min^−1^ to obtain Cu-doped porous TiO_2_ nanostructures. The Cu-doped porous TiO_2_ samples were denoted as *X*%Cu–TiO_2_, where *X* was the molar percentage of the doped Cu element. The porous TiO_2_ (TiO_2_) was synthesized by following the same procedure except for the absence of Cu(NO_3_)_2_·3H_2_O.

### XAS characterization

Synchrotron-radiation-based O K-edge, Ti L-edge and Cu L-edge XAS measurements were carried out at the MCD-A and MCD-B beamlines (Soochow Beamline for Energy Materials) (BL12B) of National Synchrotron Radiation Laboratory (NSRL) in Hefei, China. The electron beam energy of the storage ring was 800 MeV with an average stored current of 300 mA. The photo energy ranged from 100 to 1000 eV with an energy resolution of 0.2 eV. All of the data were recorded in the total electron yield (TEY) mode by collecting the sample drain current under a vacuum greater than 5 × 10^−8^ Pa. The resolving power of the grating was typically *E*/Δ*E* = 1000, and the photon flux was 5 × 10^8^ photons per second. The XAS spectra of standard anatase TiO_2_, Cu_2_O and CuO powders were collected for comparison. Ti K-edge and Cu K-edge XAS measurements were performed at the beamline 1W1B of Beijing Synchrotron Radiation Facility (BSRF) in Beijing, China. The storage ring of BSRF was operated at 2.5 GeV with the maximum stored current of 250 mA.

### SRPES characterization

SRPES experiments were performed at the Photoemission Endstation (BL10B) in the NSRL. Valence-band spectra were measured using synchrotron-radiation light as the excitation source with a photon energy of 169.50 eV and referenced to the Fermi level (*E*_F_ = 0) determined from Au. A sample bias of −10 V was applied in order to observe the secondary electron cutoff. The work function (*Φ*) was determined by the difference between the photon energy and the width of whole valence-band spectra.

### In situ DRIFTS characterization

In situ DRIFTS measurements were performed using a Bruker IFS 66v Fourier-transform spectrometer equipped with a Harrick diffuse reflectance accessory at the Infrared Spectroscopy and Microspectroscopy Endstation (BL01B) of NSRL. Each spectrum was recorded by averaging 256 scans at a resolution of 4 cm^−1^. The samples were placed in an infrared (IR) reaction chamber sealed with ZnSe windows, which is specifically designed to examine highly scattered powder samples in diffuse reflection mode. After sample loading, the chamber was purged with argon gas (99.999%) for 30 min. Then the spectrum was collected as background spectrum. During the in situ characterization, pure CH_4_ gas (99.999%) was continually introduced into the chamber.

### Photocatalytic methane halogenation

Photocatalytic methane halogenation experiments were performed in CH_4_ atmosphere at room temperature using sodium chloride as halogenation agent. Typically, 100 mg sample and specific amounts of NaCl (e.g., 10 mg, 20 mg) were dispersed in 5 mL water in a home-made quartz reactor with a diameter of 6 cm. The sample was sonicated for 5 min to form a uniform suspension and transferred in an oven at 80 °C for 5 h. After drying, 100 µL water was dropped into the notch on the reactor. The reactor was purged with CH_4_ (99.999%) for 30 min and sealed with rubber seals. Then the reactor was irradiated by a 300 W xenon lamp (PLS-SXE300, Perfect light) with a lighting area of 28.26 cm^2^. After irradiation for 4 h, the reacted gas was detected by gas chromatography (GC, 7890B, Ar carrier, Agilent) equipped with a methanation reactor (i.e., a nickel catalyst tube), a flame ionization detector (FID) and a thermal conductivity detector (TCD) for the determination of methane halides, hydrocarbons, H_2_, CO and CO_2_.

### Computational method

Spin-polarized calculations were performed with density functional theory (DFT) methods implemented in Vienna ab initio simulation package (VASP)^47,48^. Perdew, Burke and Ernzerhof (PBE)^49^ for the exchange–correlation functional of generalized gradient approximation and a plane wave cutoff of 500 eV were used. The Grimme’s DFT-D3 scheme^50^ was adopted to describe the long-range Van der Waals (vdW) interactions. In the structural optimizations, a limited number of top layers were allowed to relax with the conjugate gradient method until the residual forces were less than 0.03 eV Å^−1^. The energy convergence was 10^−5^ eV. The tetragonal anatase titanium dioxide (TiO_2_) bulk lattice parameters used in all calculations were *a* = *b* = 3.817, c = 9.575 Å and α = 90°, β = 90°, γ = 90° according to experimental results. A slab model containing 1 × 3 × 1 unit cells of anatase (101) surface was adopted to simulate key intermediates adsorption behaviors on anatase surface. The thickness of vacuum layer was 20 Å in a direction perpendicular to the surface to avoid the interaction between two neighboring slabs. The first Brillouin zone was sampled with 1 × 1 × 1 k-points both for structure optimization. The adsorption energy of CH_4_* (*E*_ads_) was calculated based on the following formulas: *E*_ads_(CH_4_*) = *E*(CH_4_*) − *E*(*) − *E*(CH_4_). *E*(*) and *E*(CH_4_*) were the total energies of anatase(101) surface or Cu(copper) doped anatase(101) surface, and CH_4_* adsorbed surface.

### Reporting summary

Further information on research design is available in the Nature Portfolio Reporting Summary linked to this article.

## Supplementary information

Supplementary Information

Reporting Summary

## Data Availability

All data that support the findings in this paper are available within the article and its Supplementary Information or are available from the corresponding authors upon reasonable request. Source data for the following figures are provided with this paper. Figures 2a–f, 3a–c, Extended Data Fig. 1e–g, Fig. S3a–e, Fig. S4, Fig. S5, Fig. S6a, b, Fig. S7, Fig. S9a, b, Fig. S10a–c, Fig. S11a, b, Fig. S13, Fig. S14a, b, Fig. S15a, b, Fig. S16a, b, Fig. S17a, b, Fig. S18, Fig. S19, Fig. S20a, b, Fig. S21d–f, Fig. S22, Fig. S23a, b, Fig. S24a, b, Fig. S26, Fig. S32, Fig. S32, Fig. S33, Fig. S34, Fig. S35, Fig. S36. Source data are provided with this paper.

## Source data

Source Data
