# Supplementary material for: Sustainable methane utilization technology via photocatalytic halogenation with alkali halides
Source: Nat Commun. 2023 Mar 14;14:1410. doi: 10.1038/s41467-023-36977-0 (PMC10014990; doi:10.1038/s41467-023-36977-0)
Supplement: Supplementary file 1 — Supplementary Information [file 41467_2023_36977_MOESM1_ESM.pdf]

Supplementary Information

Sustainable methane utilization technology via photocatalytic halogenation with alkali halides

Jun Ma[1,2,3,¶], Can Zhu[4,¶], Keke Mao[5,¶], Wenbin Jiang[1], Jingxiang Low[1], Delong Duan[1], Huanxin Ju[6], Dong Liu[1,2], Kun Wang[4], Yijing Zang[7,8], Shuangming Chen[1], Hui Zhang[8], Zeming Qi[1], Ran Long[1]*, Zhi Liu[7,8], Li Song[1], and Yujie Xiong[1,3,9]*

[1]School of Chemistry and Materials Science, National Synchrotron Radiation Laboratory, School of Nuclear Science and Technology, University of Science and Technology of China, Hefei, Anhui 230026, China
[2]Suzhou Institute for Advanced Research, University of Science and Technology of China, Suzhou, Jiangsu 215123, China
[3]Institute of Energy, Hefei Comprehensive National Science Center, 350 Shushanhu Rd., Hefei, Anhui 230031, China
[4]Department of Chemistry, Fudan University, 2005 Songhu Road, Yangpu District, Shanghai 200438, China
[5]School of Energy and Environment Science, Anhui University of Technology, Maanshan, Anhui 243032, China
[6]PHI China Analytical Laboratory, CoreTech Integrated Limited, 402 Yinfu Road, Nanjing, Jiangsu 211102, China
[7]School of Physical Science and Technology, ShanghaiTech University, Shanghai 201203, China.
[8]State Key Laboratory of Functional Materials for Informatics, Shanghai Institute of Microsystem and Information Technology, Chinese Academy of Sciences, Shanghai 200050, China.
[9]Anhui Engineering Research Center of Carbon Neutrality, College of Chemistry and Materials Science, Key Laboratory of Functional Molecular Solids, Ministry of Education, Anhui Normal University, Wuhu, Anhui 241002, China.
[¶]These authors contributed equally: Jun Ma, Can Zhu, Keke Mao.

*e-mail: longran@ustc.edu.cn; yjxiong@ustc.edu.cn

This PDF file includes:

Supplementary text
Supplementary Figs 1 to 37

Supplementary Tables 1 to 7
Supplementary references (1–9)

Experimental section

Materials

Tetra-n-butyl titanate ($Ti(OBu)_4$) (analytical grade), Pluronic F127 (MW ~ 12000) and $^{13}CH_4$ isotope (99%) were purchased from Sigma-Aldrich. Copper nitrate trihydrate ($Cu(NO_3)_2 \cdot 3H_2O$), anhydrous ethanol, hydrochloric acid (HCl, 36 wt.%) and glacial acetic acid (CH_3COOH) were purchased from Sinopharm Chemical Reagent Co., Ltd. The water used in all experiments was de-ionized (DI). All the chemical reagents were used as received without further purification.

Calculation of production rate and selectivity

The production rate of CH_3Cl from photocatalytic methane chlorination over Cu-TiO_2 was calculated according the equation:

$$\mathrm{PR}_{\mathrm{CH_3Cl}} = \frac{N(\mathrm{CH_3Cl})}{\mathrm{S} \times \mathrm{t}} \quad \text{(S1)}$$

where PR is the production rate of CH_3Cl while $N(CH_3Cl)$, S and t are the molar amount of generated CH_3Cl, irradiated area and reaction time, respectively. The molar amount of generated CH_3Cl was determined via the calibration curve of standard CH_3Cl gas. The production rate of other products was calculated according to the equation similar to that of CH_3Cl, except for the molar amounts.

The selectivity of CH_3Cl from photocatalytic methane chlorination over Cu-TiO_2 was calculated according the equation:

$$\mathrm{Selectivity}_{\mathrm{CH_3Cl}} = \frac{N(\mathrm{CH_3Cl})}{N(\mathrm{CH_3Cl}) + N(\mathrm{CO_2}) + N(\mathrm{CO}) + N(\mathrm{C_2H_6}) + N(\mathrm{C_2H_4})} \times 100\% \quad \text{(S2)}$$

where $N(CH_3Cl)$, $N(CO_2)$, $N(CO)$, $N(C_2H_6)$ and $N(C_2H_4)$ are the molar amounts of generated CH_3Cl, CO_2, CO, C_2H_6 and C_2H_4, respectively.

Calculation of AQE

The apparent quantum efficiency (AQE) was measured under the irradiation of a 254 nm or 365 nm monochromatic LED lamp with an irradiation area of 1 cm^2. The light intensity was determined by an optical power meter. The AQE was calculated according to the equation

$$\mathrm{AQE} = \frac{2\times N(\mathrm{CH_3Cl})+2\times N(\mathrm{C_2H_6})+4\times N(\mathrm{C_2H_4})}{N(\mathrm{photons})}\times 100\% \qquad \text{(S3)}$$

where $N(CH_3Cl)$, $N(C_2H_6)$ and $N(C_2H_4)$ are the amounts of generated CH_3Cl, CH_3CH_3 and CH_2CH_2, respectively, and N(photons) is the number of incident photons.

Cyclic photocatalytic methane halogenation test

The photocatalytic methane halogenation experiments were performed in a gas-solid phase reaction system, where the photocatalyst and NaCl were mixed beforehand and the water in the reactor was vaporous. As for the cyclic test, the Cu-TiO_2 catalyst was washed using water after 4 h photocatalysis. Then the catalyst was again mixed with NaCl and dried at 80 ℃. Subsequently, 100 μL water was dropped into the notch on the reactor. The reactor was purged with CH_4 (99.999%) for 30 min and sealed with rubber seals. Finally, the reactor was irradiated by a 300 W xenon lamp.

Synthesis of methyl o-toluate

In a typical procedure, 0.4 g NaOH was added into the o-methylbenzoic acid (1.36 g, 10 mmol) in dimethylformamide (DMF, 3 mL) at room temperature. The mixture was

stirred at room temperature for 30 min. The reaction flask was sealed with a rubber stopper, connected to a nitrogen balloon to maintain a constant pressure in the reaction system. Subsequently, CH_3Br (5.0 mol L^{-1} in tetrahydrofuran (THF), 4.0 mL, 20 mmol, 2.0 equiv.) was slowly injected into the reaction system at 0 ℃. After that, the reaction mixture was allowed to warm up to room temperature, and further stirred at room temperature for 18 h. H_2O (10 mL) was added to quench the reaction, and the mixture was extracted with ethyl acetate for three times (10 mL × 3). The combined organic phase was dried over anhydrous Na_2SO_4, concentrated under reduced pressure, and then purified by flash chromatography (eluent:PE/EtOAc = 50:1) to provide methyl o-toluate (1.27 g, 85% yield) as a colorless oil.

Synthesis of 1-methylindole

NaOH (0.44 g, 11 mmol, 1.1 equiv.) was added into a solution of indole (1.17 g, 10 mmol) in DMF (20 mL) at room temperature. The mixture was stirred at room temperature for 30 min. The reaction flask was sealed with a rubber stopper, connected to a nitrogen balloon to maintain a constant pressure in the reaction system. Subsequently, CH_3Br (5.0 mol L^{-1} in THF, 4.0 mL, 20 mmol, 2.0 equiv.) was slowly injected into the reaction system at 0 ℃. After that, the reaction mixture was allowed to warm up to room temperature, and further stirred at room temperature for 18 h. H_2O (25 mL) was added to quench the reaction, and the mixture was extracted with ethyl acetate for three times (25 mL × 3). The combined organic phase was dried over anhydrous Na_2SO_4, concentrated under reduced pressure, and then purified by flash chromatography (eluent: PE/EtOAc = 50:1) to provide 1-methylindole (1.26 g, 96%

yield) as a light yellow oil.

Sample characterizations

Transmission electron microscopy (TEM) images were taken on a Hitachi Model H7700 microscope at 100 kV. High-resolution TEM (HRTEM) images, scanning TEM (STEM) image and energy-dispersive X-ray spectroscopy (EDS) mapping profiles were recorded on an FEI Talos F200X field-emission high-resolution transmission electron microscope at 200 kV.

X-ray photoelectron spectroscopy (XPS) was conducted on PHI 5000 VersaProbe III using a monochromatic Al Kα X-ray source under the high power mode with an analysis area of 1400 μm × 100 μm, where the X-ray beam of 100 μm was scanned to reduce the X-ray-induced reduction of Cu ions.

Powder X-ray diffraction (XRD) patterns were recorded by using a Philips X'Pert Pro Super X-ray diffractometer with Cu-Kα radiation (λ = 1.54178 Å).

Nitrogen adsorption-desorption isotherms were measured with a Micromeritics ASAP 2020 adsorption apparatus at 77 K to 1 bar.

UV–vis–NIR diffuse reflectance spectra were recorded in the spectral region of 250- 1200 nm with a Shimadzu SolidSpec-3700 spectrophotometer.

EPR spectra were collected using a JEOL JES-FA200 electron spin resonance spectrometer at room temperature (9.062 GHz).

Ambient pressure XPS (AP-XPS) spectra were collected at the beamline BL02B1 of Shanghai Synchrotron Radiation Facility (SSRF). The light irradiation was introduced into the analysis chamber through an observation window using a 300 W

xenon lamp.

Supplementary Figures

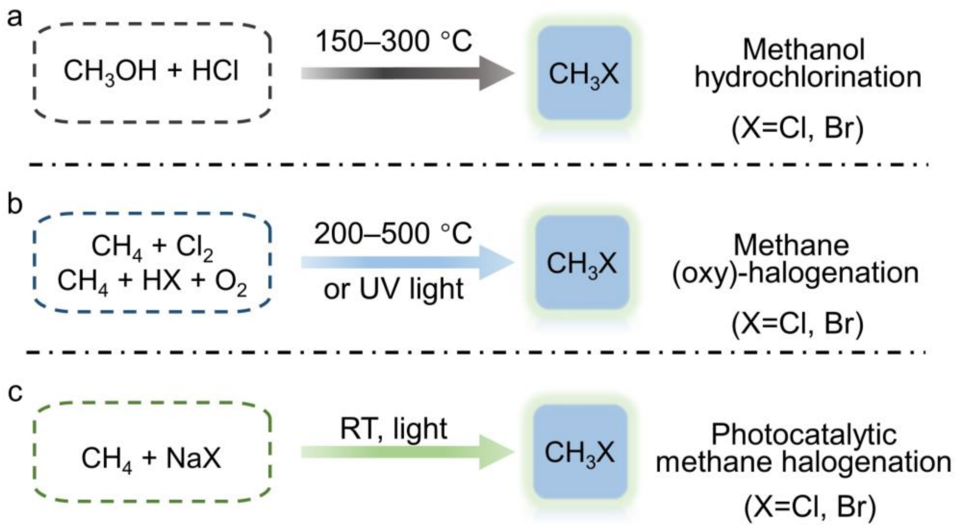

Supplementary Fig. 1 | (**a**) Traditional methanol hydrochlorination approach, (**b**) methane (oxy)-halogenation and (**c**) sustainable photocatalytic methane halogenation using alkali halides for methyl halide production.

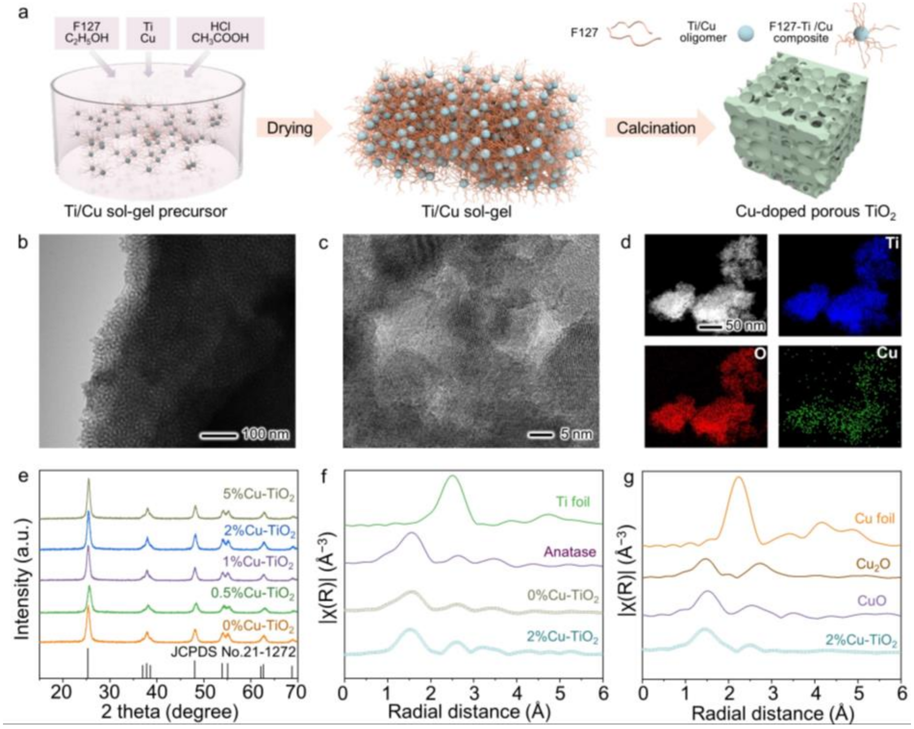

Supplementary Fig. 2 | Synthesis and characterization of Cu-doped porous TiO_2 nanostructures. (**a**) Scheme illustration of preparation procedure for *X*%Cu-TiO_2. (**b**) TEM and (**c**) high-resolution TEM (HRTEM) images of 2%Cu-TiO_2. (**d**) Scanning TEM (STEM) image and EDS elemental mapping profiles of 2%Cu-TiO_2 with Ti (blue), O (red), and Cu (green) distribution. (**e**) Powder XRD patterns of as-prepared porous *X*%Cu-TiO_2 nanostructures. (**f**) k^3-weighted Fourier-transform Ti K-edge and (**g**) Cu K-edge EXAFS spectra of 0%Cu-TiO_2 and 2%Cu-TiO_2.

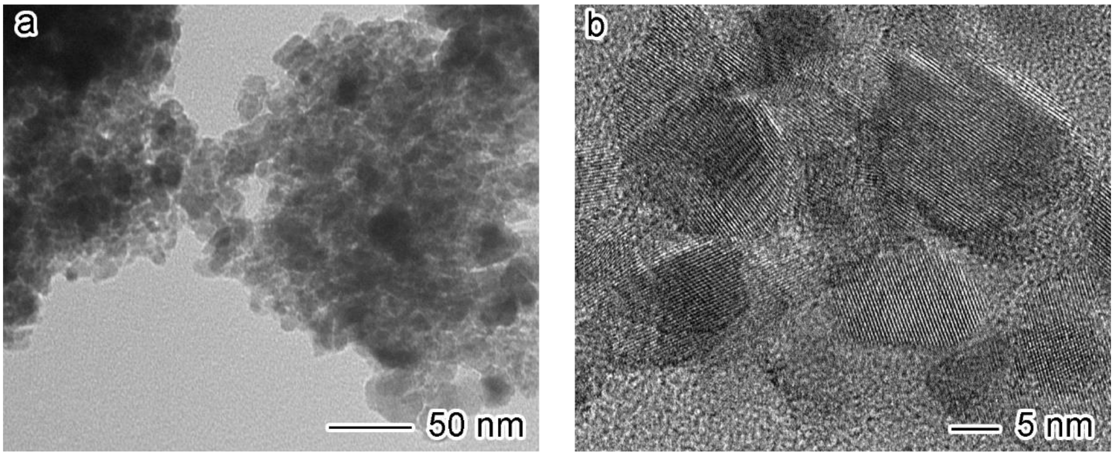

Supplementary Fig. 3 | (**a**) TEM and (**b**) HRTEM images of 0%Cu-TiO_2.

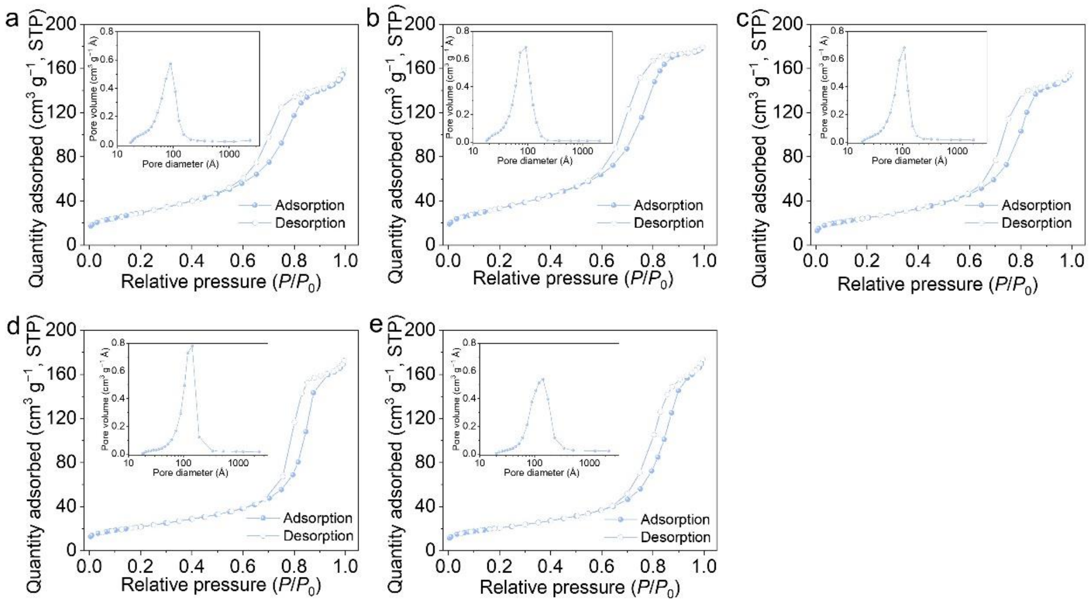

Supplementary Fig. 4 | Nitrogen adsorption–desorption isotherms for (**a**) 0%Cu-TiO_2, (**b**) 0.5%Cu-TiO_2, (**c**) 1%Cu-TiO_2, (**d**) 2%Cu-TiO_2, and (**e**) 5%Cu-TiO_2 at 77 K. The insets show the pore size distribution of the samples.

The nitrogen adsorption–desorption isotherms are obtained to determine the Brunauer–Emmet–Teller (BET) surface area and the pore size of the *X*%Cu-TiO_2. The BET surface areas (Supplementary Table 1) are calculated according to nitrogen adsorption-desorption isotherms at 77 K (Supplementary Fig. 4a–e).

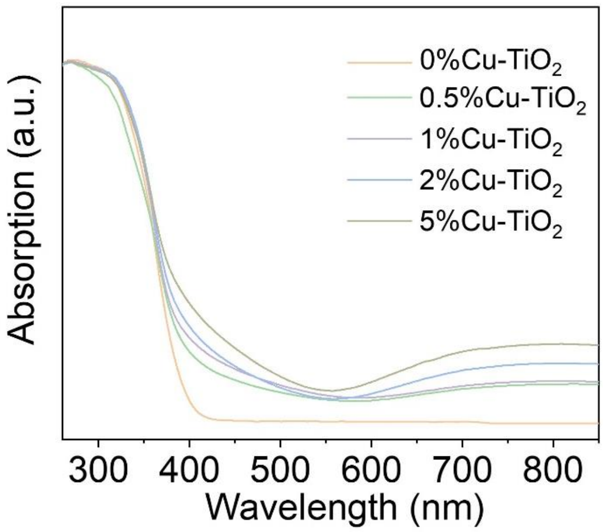

Supplementary Fig. 5 | UV–vis–NIR diffuse reflectance spectra of the prepared *X*%Cu-TiO_2.

Based on UV–vis diffuse reflectance spectra of the *X*%Cu-TiO_2 (Supplementary Fig. 5), we observe that light absorption edges of *X*%Cu-TiO_2 red-shift as the doped Cu increases due to the presence of V_O and $2E_g \rightarrow 2T_{2g}$ transitions from O to Cu atoms. Moreover, *X*%Cu-TiO_2 exhibits increased absorption in the vis–NIR region attributed to d–d transitions of the Cu dopants.[1]

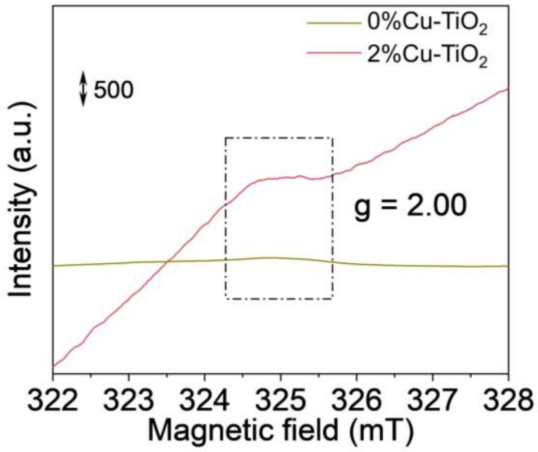

Supplementary Fig. 6 | The electron paramagnetic spectroscopy (EPR) of 0%Cu-TiO_2 and 2%Cu-TiO_2.

As shown in Supplementary Fig. 6, the EPR signals at g = 2.00 can be assigned to oxygen vacancies (V_O), suggesting the enhanced V_O concentration on the TiO_2 after Cu doping. Notably, the signal intensity for Cu^{2+} in the Cu-TiO_2 is substantially higher than that for oxygen vacancies. Moreover, the characteristic EPR spectrum of Cu^{2+} exhibits a broad signal.[2,3] For this reason, the EPR characterization in Supplementary Fig. 6 is conducted at a narrow range of magnetic field (322–328 mT) to reduce the interference of Cu^{2+} and effectually detect the oxygen vacancies.

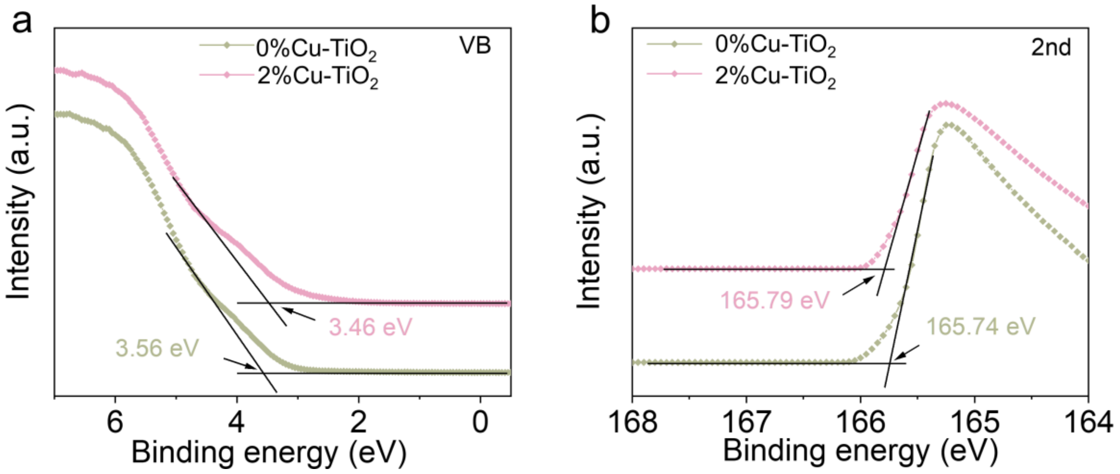

Supplementary Fig. 7 | (**a**) Valence band (VB) spectra and (**b**) secondary electron cutoff (E_{cutoff}) of 0%Cu-TiO_2 and 2%Cu-TiO_2.

Valence band (VB) spectra are acquired using synchrotron-radiation light as the excitation source with photon energy of 169.50 eV and referenced to the Fermi level (E_F = 0) determined from Au. The VB spectra (Supplementary Fig. 7a) show that the valence band maxima (VBMs) of 0%Cu-TiO_2 and 2%Cu-TiO_2 are 3.46 and 3.56 eV below E_F, respectively. A sample bias of −10 V is applied to observe the secondary electron cutoff (2nd). The work functions (Φ) of 0%Cu-TiO_2 and 2%Cu-TiO_2, which represent the location of Fermi level (E_F) versus vacuum level ($E_{vac.}$) by second electron cutoff (Supplementary Fig. 7b), turn out to be 3.76 and 3.71 eV, respectively. On the basis of the results above, the VBM of 0%Cu-TiO_2 and 2%Cu-TiO_2 can be estimated to be at 7.32 and 7.17 eV correlated to the vacuum level, respectively.

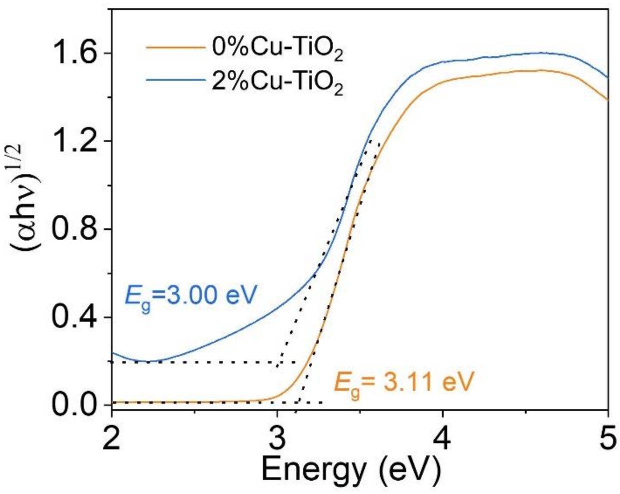

Supplementary Fig. 8 | Determination of optical band gaps (E_g) of 0%Cu-TiO_2 and 2%Cu-TiO_2.

According to Kubelka–Munk functions, the band gaps (E_g) of 0%Cu-TiO_2 and 2%Cu-TiO_2 are determined to be 3.11 and 3.00 eV, respectively.

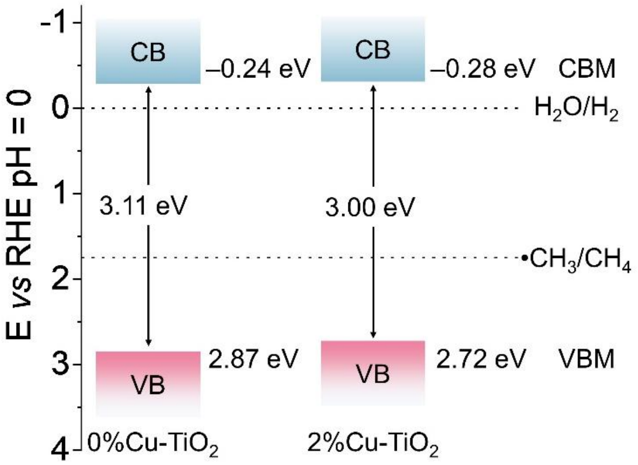

Supplementary Fig. 9 | Schematic illustration for the electronic band structures of 0%Cu-TiO_2 and 2%Cu-TiO_2.

Taking the determined work functions and band gaps (E_g) together, the conduction band minimums (CBM) can be determined to be 4.21 and 4.17 eV versus vacuum level for 0%Cu-TiO_2 and 2%Cu-TiO_2, respectively, which are also −0.24 and −0.28 eV correlated to the reversible hydrogen electrode (RHE). As a result, the electronic structures versus RHE can be demonstrated in Supplementary Fig. 9.

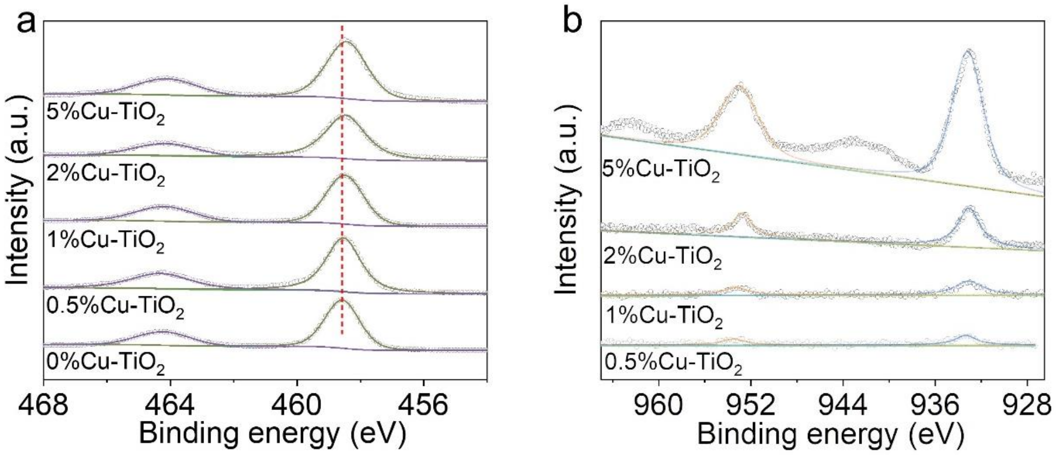

Supplementary Fig. 10 | High-resolution (**a**) Ti 2*p* and (**b**) Cu 2*p* XPS spectra for *X*%Cu-TiO_2.

The XPS is collected to reveal the chemical status of *X*%Cu-TiO_2. For the high-resolution Ti 2*p* XPS spectra (Supplementary Fig. 10a), two main binding energy peaks respectively originating from $2p_{3/2}$ and $2p_{1/2}$ signals due to spin-orbit coupling can be found on all the prepared *X*%Cu-TiO_2 samples. Moreover, the binding energy shifts to a lower value as the doping Cu content increases, due to the increase in oxygen vacancy concentrations. Moreover, Supplementary Fig. 10b shows that two XPS peaks are located at 933.14 and 953.04 eV, indicating the existence of Cu^{2+} species.

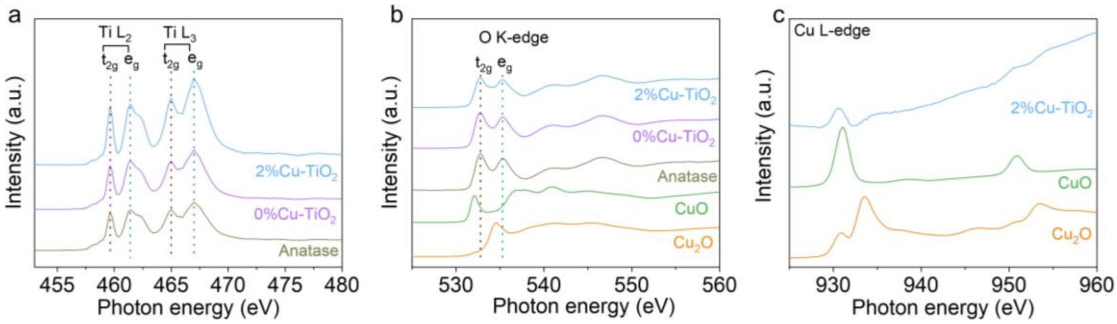

Supplementary Fig. 11 | (**a**) Ti L-edge, (**b**) O K-edge, and (**c**) Cu L-edge with soft X-ray excitation for 0%Cu-TiO_2, 2%Cu-TiO_2 and reference samples.

X-ray absorption spectroscopy (XAS) with soft X-ray excitation is used to probe the electronic structure of the *X*%Cu-TiO_2. For the as-synthesized *X*%Cu-TiO_2, the Ti L-edge and O K-edge XAS spectra show typical anatase TiO_2 spectral profiles (Supplementary Fig. 11a,b), whereas the Cu L-edge XAS spectrum displays a CuO-like profile (Supplementary Fig. 11c). The hybridization between Cu and O resembles that of CuO, suggesting a +2 oxidation state for Cu associated with oxygen vacancies in the TiO_2 crystal.

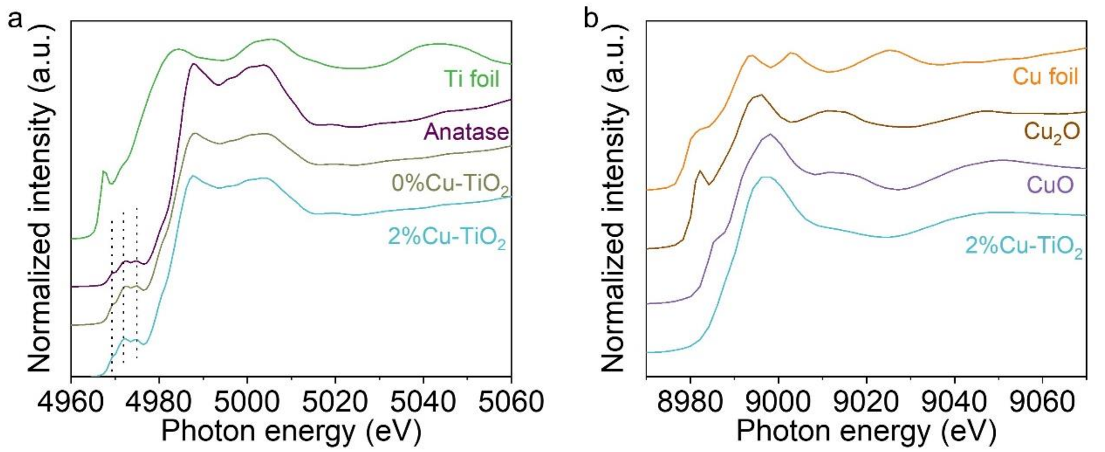

Supplementary Fig. 12 | Normalized (**a**) Ti K-edge and (**b**) Cu K-edge XANES spectra of 0%Cu-TiO_2, 2%Cu-TiO_2, and reference (Cu foil).

Supplementary Fig. 12 shows the Ti and Cu K-edge X-ray absorption near-edge structure (XANES) spectra of 0%Cu-TiO_2 and 2%Cu-TiO_2 in reference to standard Ti foil, TiO_2 (anatase), and Cu foil, Cu_2O and CuO, respectively. Ti K-edge X-ray absorption near-edge structure (XANES) spectra for 0%Cu-TiO_2, 2%Cu-TiO_2 (Supplementary Fig. 12a) possess three typical pre-edge peaks associated with the anatase phase,[4,5] corresponding to quadruple-allowed $1s \rightarrow 3d$ transitions, providing further evidence for the formation of pure anatase phase. Moreover, the Cu K-edge XANES spectrum for 2%Cu-TiO_2 (Supplementary Fig. 12b) possesses an absorption edge energy at 8996.8 eV, attributed to the characteristic absorption of Cu^{2+}.

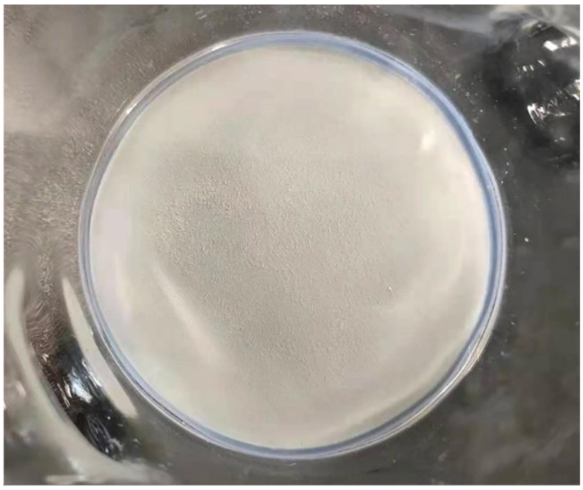

Supplementary Fig. 13 | Optical image of *X*%Cu-TiO_2 samples on the bottom of the reactor.

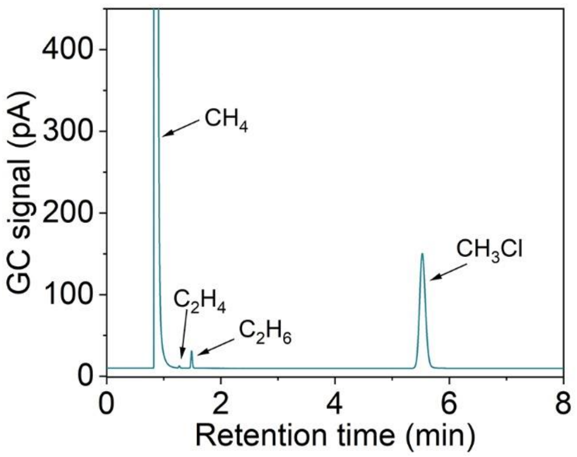

Supplementary Fig. 14 | The typical gas chromatograph (GC) traces of methane (CH_4), ethylene (C_2H_4), ethane (C_2H_6) and methyl chloride (CH_3Cl).

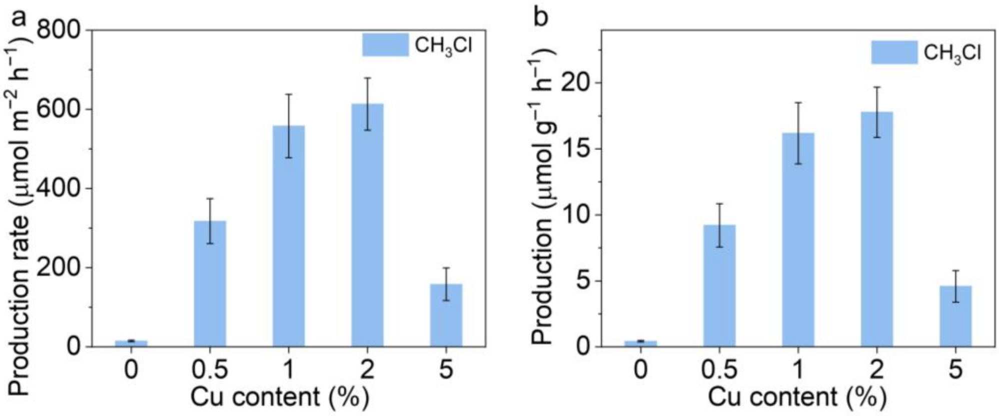

Supplementary Fig. 15 | Photocatalytic production rates of CH_3Cl from halogenation of methane by *X*%Cu-TiO_2 in terms of (**a**) catalyst loading area and (**b**) catalyst weight. (Error bars indicate standard deviations.)

As determined by gas chromatography, CH_3Cl, C_2H_4, C_2H_6, CO and CO_2 are the main products from photocatalytic halogenation of methane over *X*%Cu-TiO_2. The methane conversion over 2%Cu-TiO_2 is approximately 2.4% for 28 h. It is worth noting that, as compared with thermocatalytic methane halogenation using halogens or hydrogen halides, photocatalytic methane halogenation using alkali halides exhibits lower conversion and productivity (Supplementary Table 6). However, photocatalytic methane halogenation process has the advantages given the use of solar energy as power input and alkali halides as halogen source.

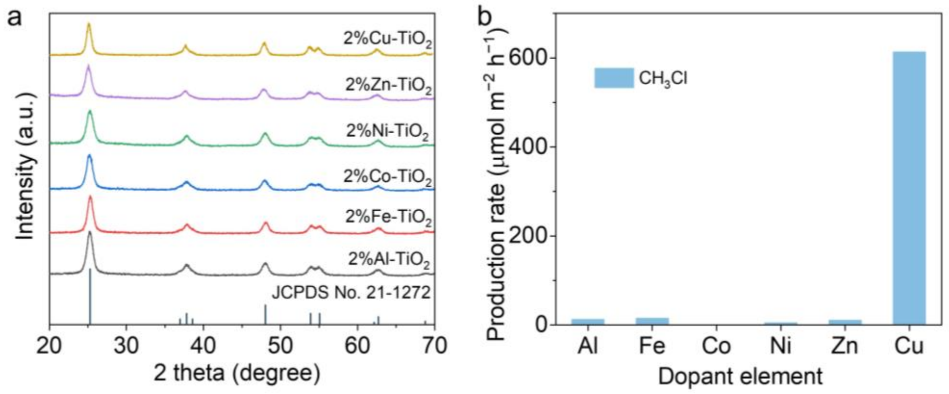

Supplementary Fig. 16 | (**a**) The XRD patterns of different element-doped TiO_2 nanostructures, including Al, Fe, Co, Ni, Zn and Cu-doped TiO_2. (**b**) The comparison of photocatalytic methane halogenation performance over Cu-TiO_2 with different element-doped TiO_2 (Al, Fe, Co, Ni, and Zn-doped TiO_2) under the same reaction conditions.

The different element-doped TiO_2 nanostructures, including Al-TiO_2, Zn-TiO_2, Fe-TiO_2, Co-TiO_2 and Ni-TiO_2, are prepared through the same method as Cu-TiO_2. As shown in Supplementary Fig. 16, these catalysts exhibit negligible activity on photocatalytic methane halogenation compared with Cu-TiO_2 catalyst, which further demonstrates the essential role of Cu in photocatalytic methane halogenation over Cu-TiO_2.

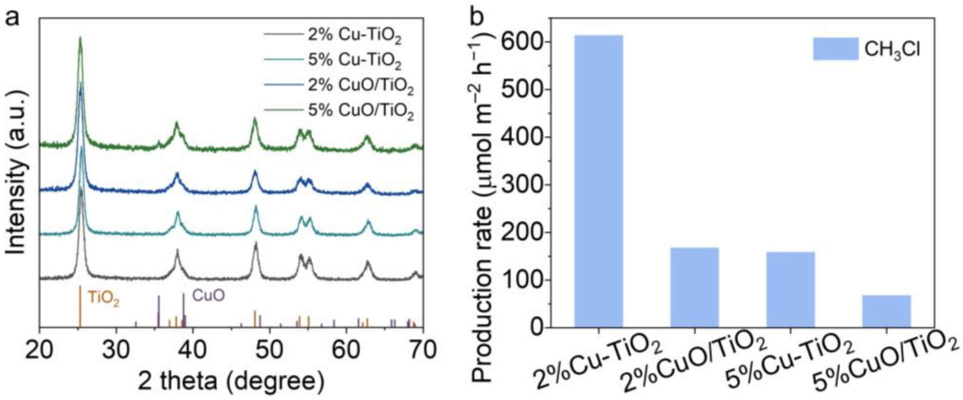

Supplementary Fig. 17 | (a) The XRD patterns of Cu-doped TiO_2 nanostructures (2%Cu-TiO_2 and 5%Cu-TiO_2) and CuO-loaded TiO_2 nanostructures (2%CuO/TiO_2 and 5%CuO/TiO_2). (**b**) Photocatalytic production rates of CH_3Cl via methane halogenation over different catalysts.

As shown in Supplementary Fig. 17a, CuO phase is observed in the XRD pattern of 5%CuO/TiO_2 (loaded), which cannot be found in the XRD pattern of 5%Cu-TiO_2. Moreover, the photocatalytic performance over the CuO/TiO_2 (loaded) is far lower than that of the Cu-TiO_2 (doped) (Supplementary Fig. 17b), indicating the significant role of Cu doping in methane and halide activation which induces oxygen vacancies with localized charge trapping centers on TiO_2.

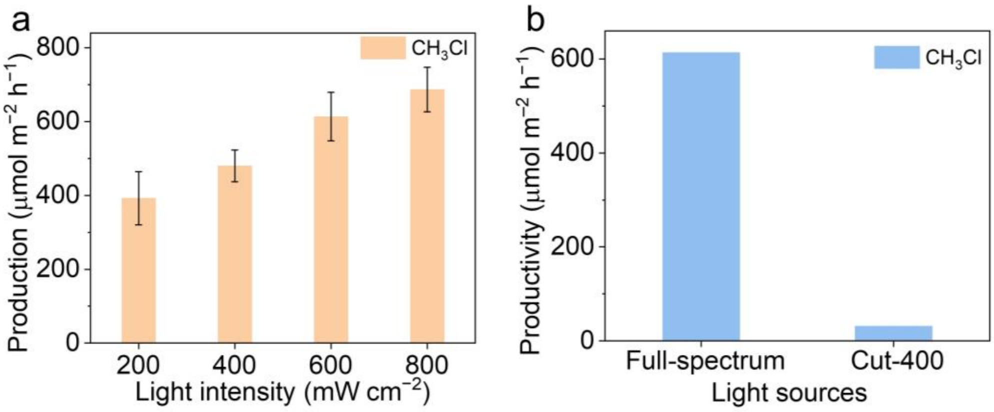

Supplementary Fig. 18 | (**a**) Photocatalytic CH_3Cl production rates over 2%Cu-TiO_2 at different light intensities. (Error bars indicate standard deviations.) (**b**) Photocatalytic CH_3Cl production rates over 2%Cu-TiO_2 under full-spectrum and cut-400 light irradiation.

The relationship between the performance of photocatalytic CH_4 halogenation using NaCl as halogenation agent and the intensity of incident light is also observed (Supplementary Fig. 18a). The production rate of CH_3Cl is promoted from 418.3 μmol h^{-1} m^{-2} to 686.8 μmol h^{-1} m^{-2} as the intensity of incident light increases from 200 to 800 mW cm^{-2}, implying that photocatalytic methane halogenation is highly related to the number of incident photons. Notably, since photocatalytic methane halogenation reaction system is a complex process, the production rate of CH_3Cl is dependent on the activation of CH_4 molecule and NaCl, which is highly related to migration efficiency of photogenerated carriers and reactants. Thus the photocatalytic CH_3Cl production rate is not linearly promoted as the light intensity increases. As shown in Supplementary Fig. 18b, the CH_3Cl production rate over 2%Cu-TiO_2 under cut-400 light irradiation is ca. 31.5 μmol m^{-2} h^{-1}.

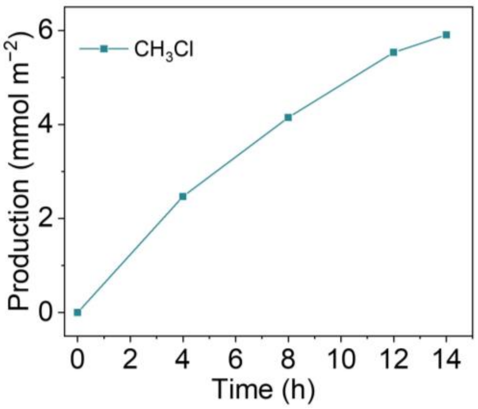

Supplementary Fig. 19 | Time-dependent photocatalytic CH_3Cl production over 2%Cu-TiO_2.

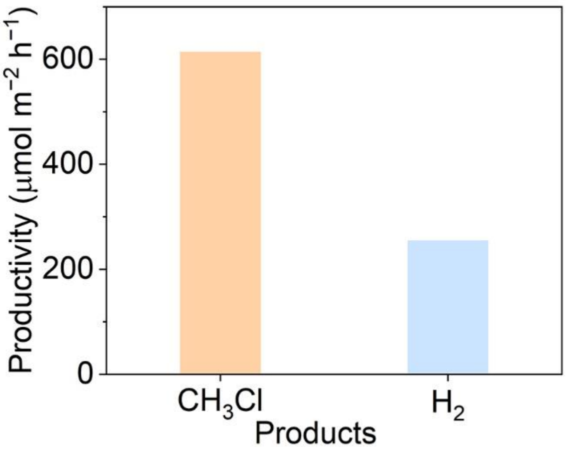

Supplementary Fig. 20 | H_2 production during the photocatalytic methane chlorination.

The amount of produced H_2 is nonstoichiometric to the amount of methyl chloride (Supplementary Fig. 20), suggesting the possibility of consuming lattice oxygen in TiO_2 during photocatalytic methane chlorination. The reaction for photocatalytic chlorination of methane is shown below:

$$CH_4 + NaCl + H_2O \rightarrow CH_3Cl + H_2 + NaOH \quad (S4)$$

$$CH_4 + NaCl + TiO_2 \rightarrow CH_3Cl + NaO_{lattice}H \quad (S5)$$

H_2 could be generated during photocatalytic methane chlorination, while the amount of H_2 is nonstoichiometric to the amount of methyl chloride (Supplementary Fig. 20), which results from probable consumption of lattice oxygen in TiO_2 during photocatalytic CH_4 chlorination. The recovery of consumed lattice oxygen can be easily conducted in the air to regenerate the pristine structure of *X*%Cu-TiO_2 (Supplementary Fig. 22).

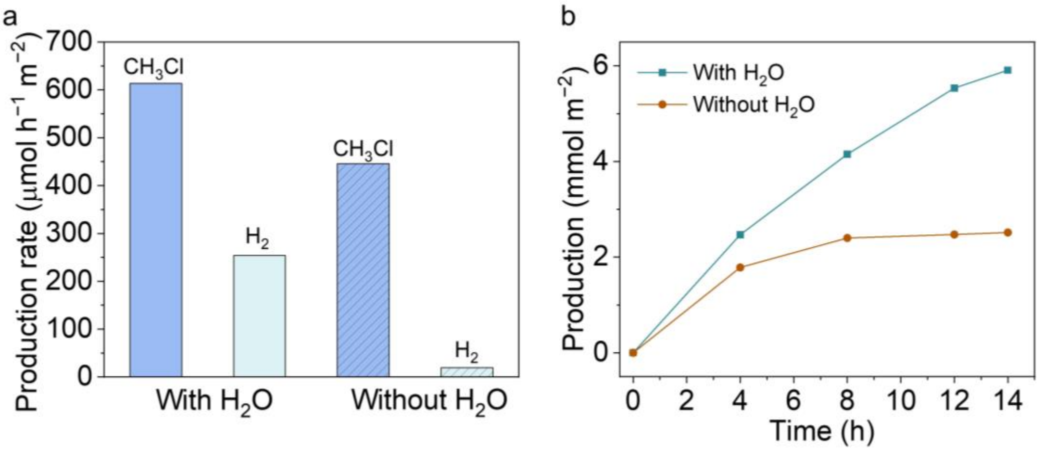

Supplementary Fig. 21 | (**a**) Photocatalytic CH_3Cl and H_2 production rates over 2%Cu-TiO_2 in the presence or absence of H_2O. (**b**) Time-dependent photocatalytic CH_3Cl production over 2%Cu-TiO_2 in the presence or absence of H_2O.

As shown in Supplementary Fig. 21, photocatalytic methane halogenation in the absence of H_2O exhibits a substantially lower production rate of CH_3Cl with a trace amount of H_2 production. Moreover, the CH_3Cl production almost stops beyond about 8 h due to the rapid consumption of lattice oxygen, which is significantly faster than that in the presence of H_2O. This is because the photogenerated electrons cannot be efficiency utilized when H_2O is absent in the system, and in turn these photogenerated electrons will consume the lattice oxygen to generate NaOH during the reaction. The consumption of lattice oxygen eventually causes the decay in photocatalytic performance of Cu-TiO_2 (Supplementary Fig. 21b). In contrast, the presence of H_2O can ease the consumption of lattice oxygen in TiO_2, by providing hydrogen source for H_2 production and oxygen source for regeneration of lattice oxygen.

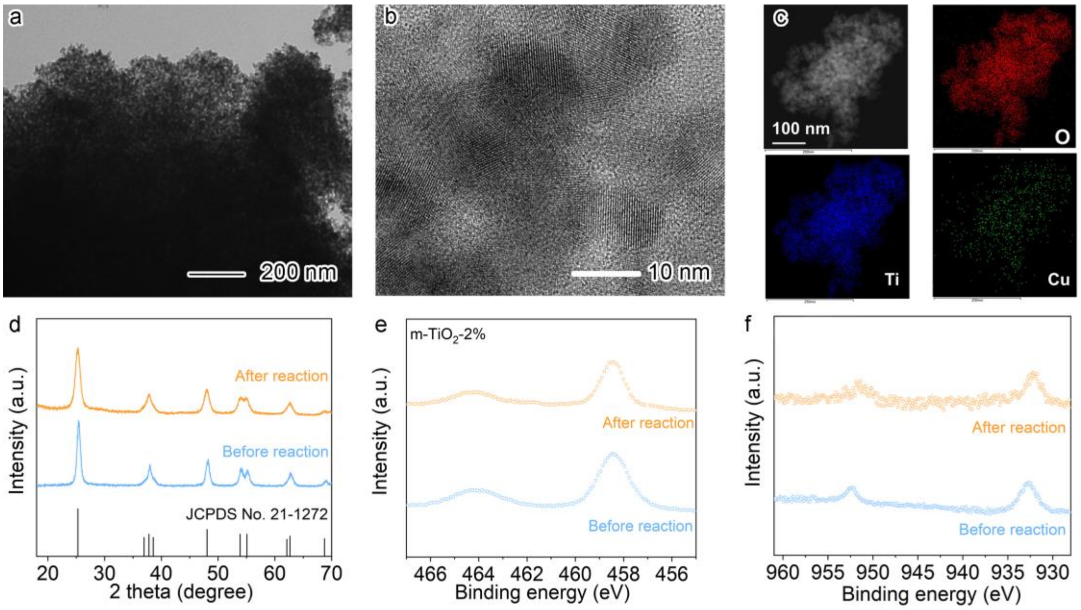

Supplementary Fig. 22 | (**a**) TEM image, (**b**) HRTEM and (**c**) STEM image and EDS elemental mapping profiles of 2%Cu-TiO_2 with Ti (blue), O (red), and Cu (green) distribution after photocatalytic reaction. (**d**) XRD patterns. (e,f) high-resolution Ti 2*p* (**e**) and Cu 2*p* (**f**) XPS spectra of 2%Cu-TiO_2 after six cyclic tests for photocatalytic methane conversion.

By comparing the TEM images, XRD patterns and XPS spectra of the 2%Cu-TiO_2 before and after photocatalytic methane halogenation, it can be confirmed that the morphology, crystal phase and elemental valence states of the 2%Cu-TiO_2 are well maintained, manifesting the excellent reusability and stability of the Cu-doped TiO_2 photocatalyst. According to the element mapping profiles, the Cu element is still uniformly distributed in the TiO_2 after reaction. Notably, as detected by EDS mapping, the Cu content slightly decreases after photocatalytic reaction (Supplementary Table 4), which may result from water washing during cyclic test. Nevertheless, with the slight loss of Cu element, the photocatalytic performance still remains stable over the successive three cycles.

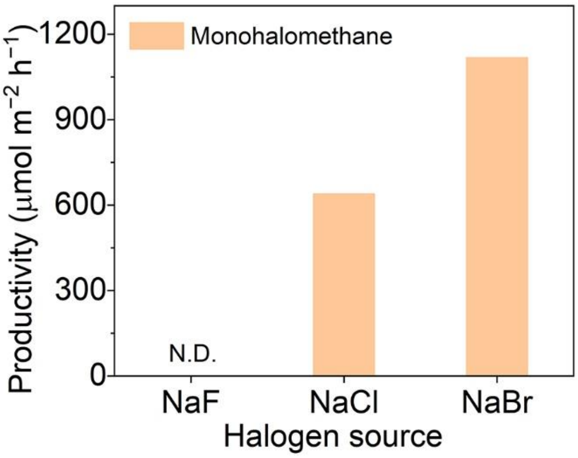

Supplementary Fig. 23 | The production rate for photocatalytic halogenation of methane using different sodium halides.

As shown in Supplementary Fig. 23, the production rate for photocatalytic halogenation of methane is greatly enhanced when NaBr is implemented as the halogen source, while no methyl halide can be detected when using NaF. These results further confirm that the photocatalytic halogenation of methane is conducted through halogen oxidation and is highly related to the electronegativity of halogen. The electronegativity values of F, Cl and Br are 4, 3 and 2.8, respectively.

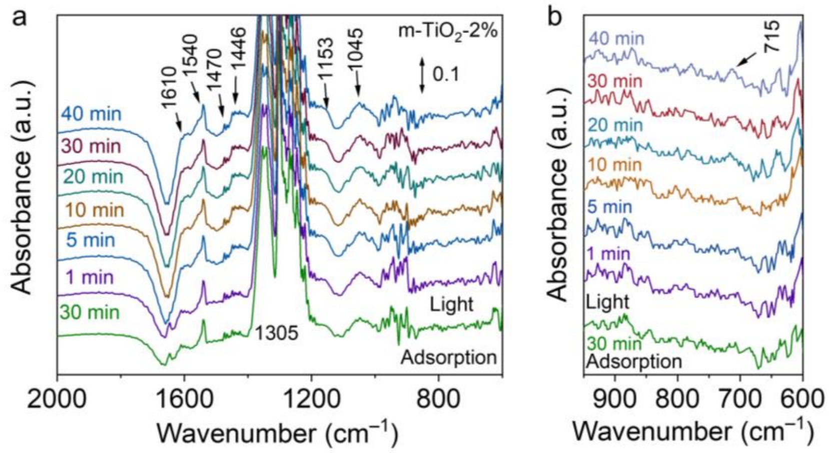

Supplementary Fig. 24 | (a) In situ DRIFTS spectra for photocatalytic methane chlorination for 2%Cu-TiO_2 with the reaction evolution under irradiation. (**b**) In situ DRIFTS spectra at the range of 600–1000 cm^{-1} for photocatalytic methane chlorination for 2%Cu-TiO_2 with the reaction evolution under irradiation.

Notably, the in situ DRIFTS spectra at the range of 600–1000 cm^{-1} are recorded to observe the formation of C–Cl bond during photocatalytic methane halogenation over 2%Cu-TiO_2 by changing the windows of the IR setup. As shown in Supplementary Fig. 24b, a peak at 715 cm^{-1}, assigned to C–Cl bond, can be observed and gradually grows upon light irradiation, further suggesting the generation of CH_3Cl from photocatalytic methane halogenation.

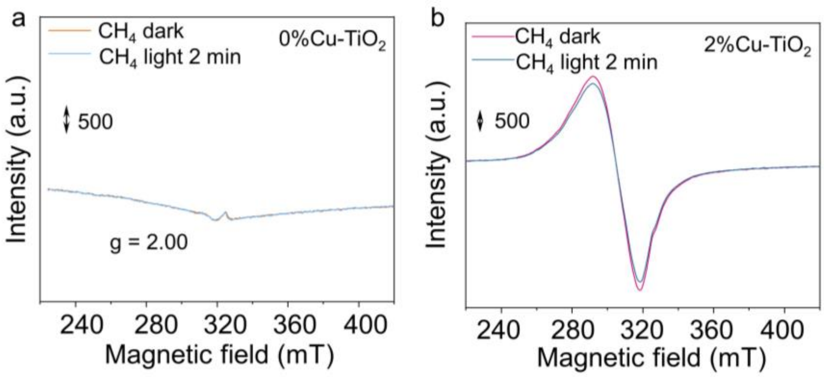

Supplementary Fig. 25 In situ EPR spectra for photocatalytic methane halogenation over (**a**) 0%Cu-TiO$_2$ and (**b**) 2%Cu-TiO$_2$ using NaCl as halogenation agent during adsorption (CH_4 dark) and photocatalytic reactions (CH_4 light).

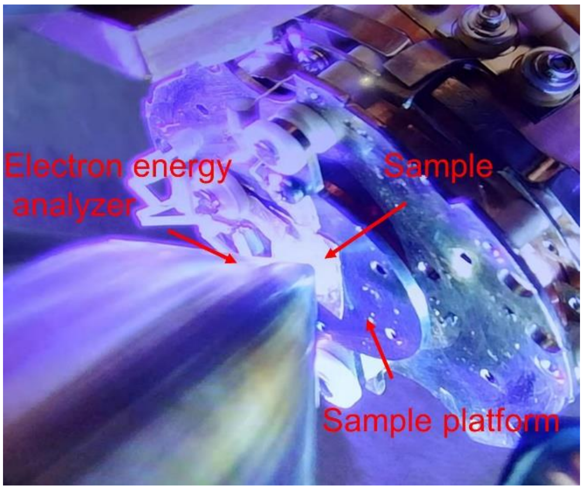

Supplementary Fig. 26 | The optical image for the AP-XPS experiment setup under light irradiation.

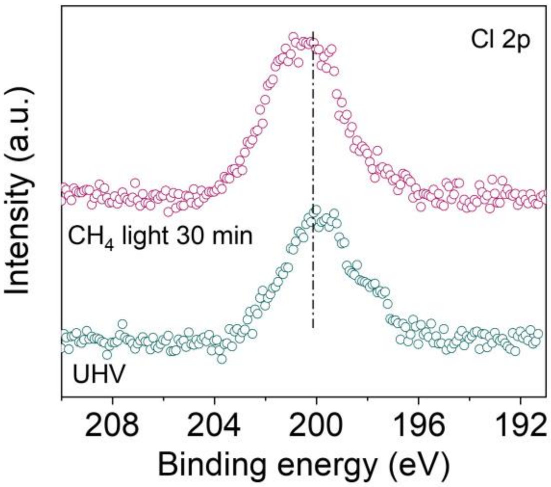

Supplementary Fig. 27 | The Cl 2*p* region of AP-XPS spectra for photocatalytic methane halogenation over 2%Cu-TiO_2 in the present of NaCl as halogenation agent under various conditions.

The AP-XPS spectra for photocatalytic methane halogenation over 2%Cu-TiO_2 in the present of NaCl as halogenation agent under dark and light irradiation conditions are shown in Supplementary Fig. 27. It is discovered that the Cl 2*p* peak becomes broader and slightly shifts toward higher binding energy upon light irradiation (Supplementary Fig. 27), indicating the oxidation of Cl of NaCl by 2%Cu-TiO_2 catalyst during the reaction. Moreover, the Cl 2*p* peak recorded in AP-XPS becomes broader and slightly shifts toward higher binding energy (Supplementary Fig. 27) because the binding energy of Cl in CH_3Cl is higher than that in NaCl. As such, the formation of C–Cl bond can be confirmed by the Cl 2*p* signal in the AP-XPS.

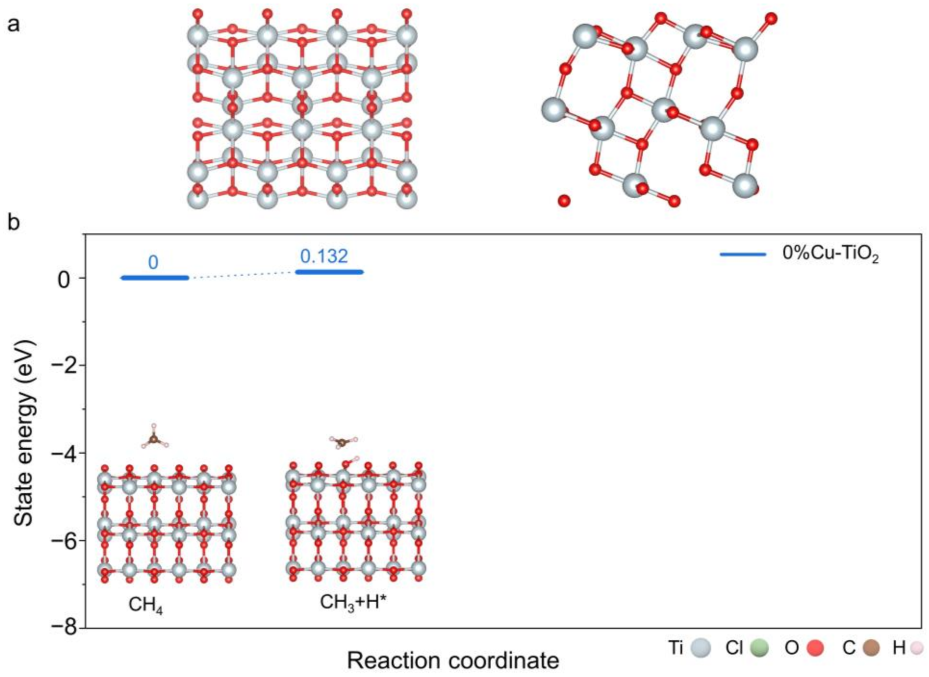

Supplementary Fig. 28 | (**a**) Top view (left) and side view (right) of the optimized structure of TiO_2 in DFT simulations. (**b**) The DFT calculations for methane adsorption on the surface TiO_2. The insets show the optimized structures for each step.

As shown in Supplementary Fig. 28, the *CH_3 intermediates can hardly be stabilized on the surface of pristine TiO_2 due to the scarcity of adsorption sites.

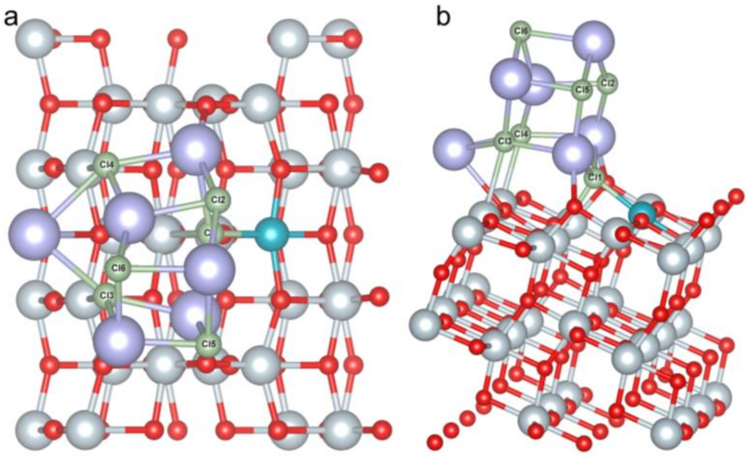

c

Atom	Charge (|e|)
Cl1	7.519
Cl2	7.785
Cl3	7.788
Cl4	7.744
Cl5	7.802
Cl6	7.781

Supplementary Fig. 29 | (**a**) Top view and (**b**) side view of the optimized structure of NaCl cluster on the surface of Cu-TiO_2 in Bader charge analysis. (**c**) The calculated charge distribution of Cl atoms for NaCl cluster on Cu-TiO_2 catalyst.

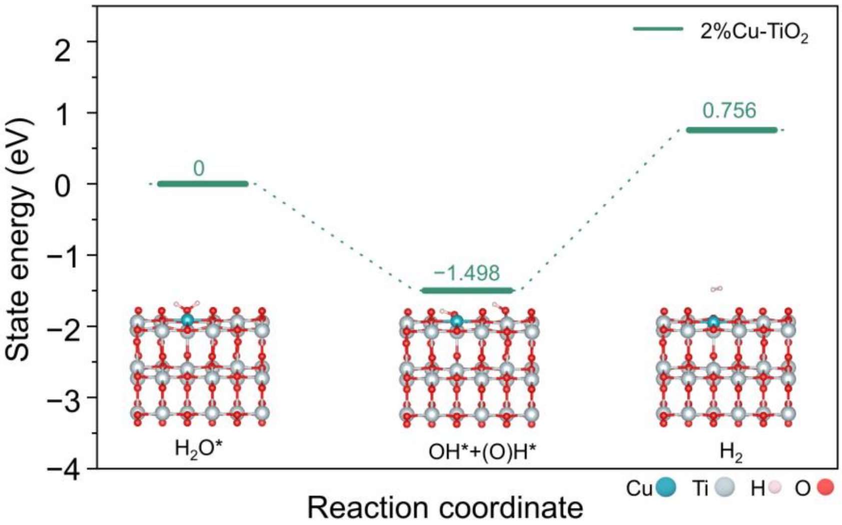

Supplementary Fig. 30 | The DFT calculations for the impact of H_2O on photocatalytic methane chlorination over Cu-TiO_2 catalyst. The insets show the optimized structures for each step.

The DFT calculations are performed to analyze the impact of water on photocatalytic methane halogenation system over Cu-TiO_2. As shown in Supplementary Fig. 30, H_2O can be first adsorbed on the oxygen vacancy, derived from the consumption of lattice oxygen during the generation of CH_3Cl and NaOH over Cu-TiO_2, which then is dissociated into OH* and (O)H* species neighboring the oxygen vacancy with an electron energy change of −1.498 eV. Upon irradiation, the H_2 can be formed through photoreduction and desorbed from the Cu-TiO_2 surface[6] while the oxygen from water will be filled into the lattice to regenerate the pristine structure of Cu-TiO_2.

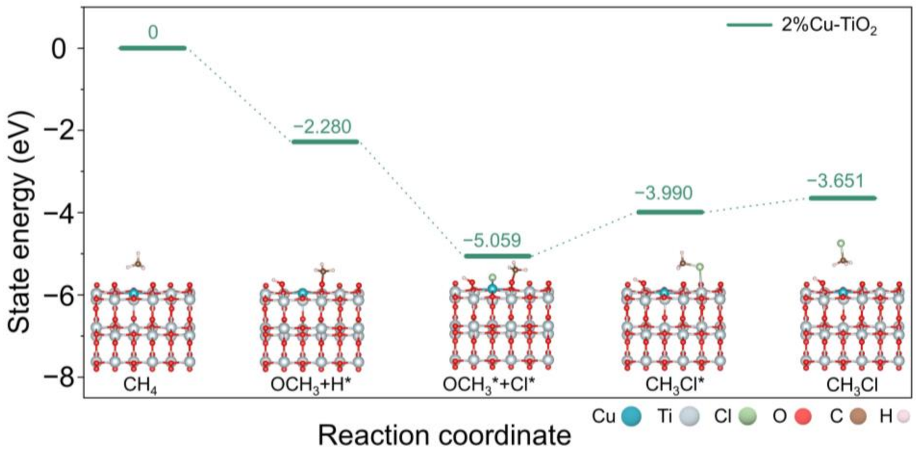

Supplementary Fig. 31 | The DFT calculations for the production of CH_3Cl in photocatalytic methane chlorination on Cu doped TiO_2 through the methoxy route. The insets show the optimized structures for each step.

Although methane molecule could be easily dissociated to form methoxy species (*O–CH_3) on the surface of Cu-doped TiO_2, the methoxy species can hardly be transformed in the following reaction due to its strong binding on the surface of Cu-doped TiO_2 with an adsorption energy of −5.059 eV.

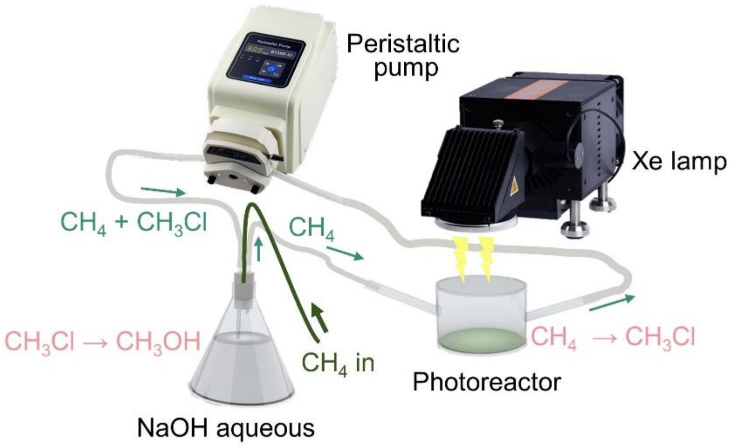

Supplementary Fig. 32 | The cycling reaction system for photocatalytic methane conversion.

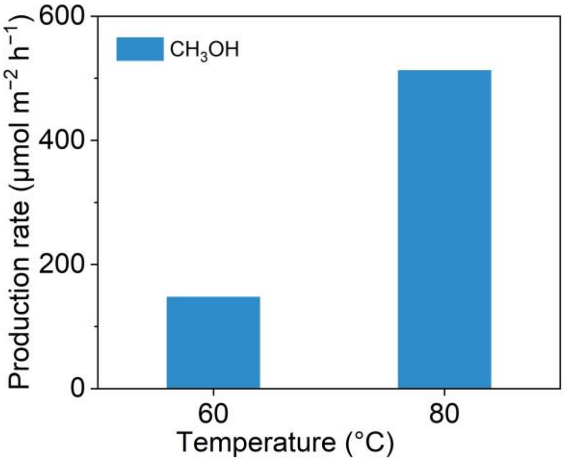

Supplementary Fig. 33 | The production rates of methanol via methyl halide intermediates in the cyclic photocatalytic methane halogenation system.

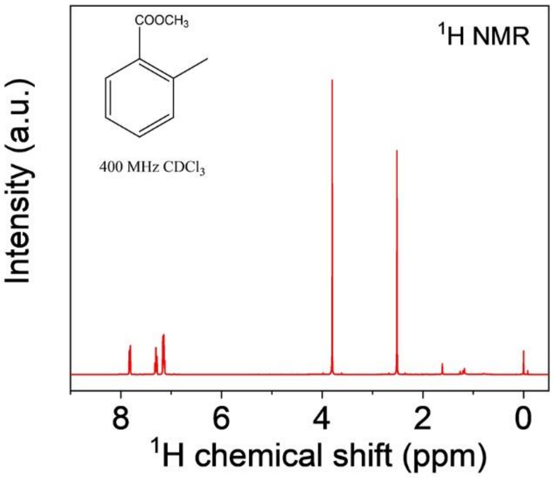

Supplementary Fig. 34 | ^{1}H NMR spectrum for the products produced from the synthesis of methyl o-toluate through CH_3Br.

Methyl o-toluate: ^{1}H NMR (400 MHz, Chloroform-d) δ 7.82 (dd, J = 8.1, 1.5 Hz, 1H), 7.30 (td, J = 7.5, 1.5 Hz, 1H), 7.22-6.97 (m, 2H), 3.80 (d, J = 0.9 Hz, 3H), 2.52 (s, 3H).

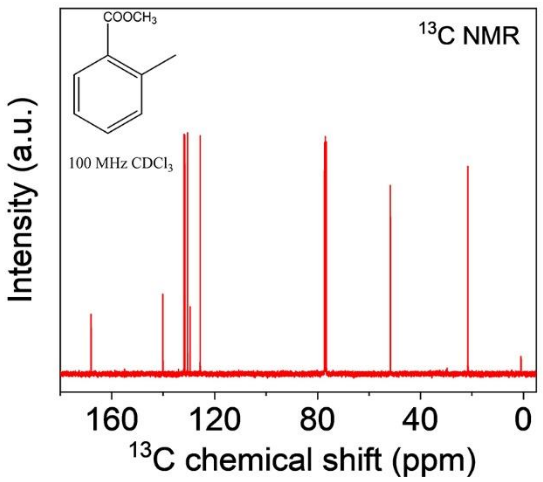

Supplementary Fig. 35 | ^{13}C NMR spectrum for the products produced from the synthesis of methyl o-toluate through CH_3Br.

Methyl o-toluate: ^{13}C NMR (100 MHz, Chloroform-d) δ 168.02, 140.11, 131.89, 131.61, 130.50, 129.51, 125.63, 51.72, 21.64.

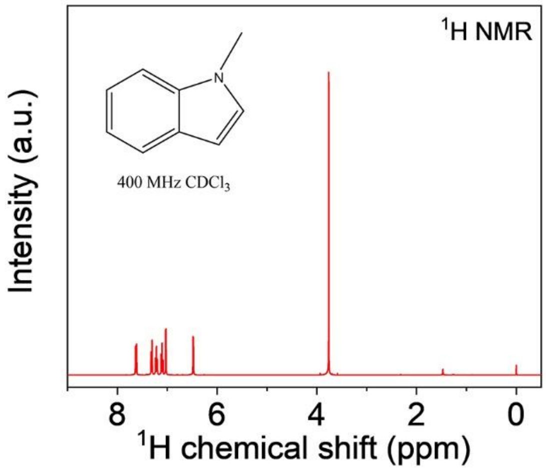

Supplementary Fig. 36 | ¹H NMR spectrum for the products produced from the synthesis of 1-methylindole through CH_3Br.

1-methylindole: ¹H NMR (400 MHz, Chloroform-d) δ 7.62 (d, J = 7.9 Hz, 1H), 7.31 (dd, J = 8.2, 1.0 Hz, 1H), 7.26-7.18 (m, 1H), 7.16-7.07 (m, 1H), 7.03 (d, J = 3.1 Hz, 1H), 6.48 (dd, J = 3.2, 0.8 Hz, 1H), 3.76 (s, 3H).

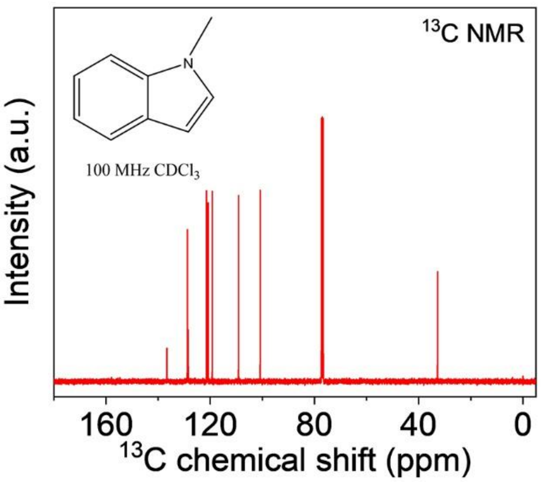

Supplementary Fig. 37 | ^{13}C NMR spectrum for the products produced from the synthesis of 1-methylindole through CH_3Br.

1-methylindole: ^{13}C NMR (100 MHz, Chloroform-d) δ 136.64, 128.73, 128.42, 121.43, 120.81, 119.21, 109.13, 100.83, 32.75.

Supplementary Tables

Supplementary Table 1 | Calculated BET surface area of the prepared samples.

Sample	BET surface area ($m^2\ g^{-1}$)
0%Cu-TiO_2	106.26
0.5%Cu-TiO_2	120.00
1%Cu-TiO_2	88.55
2%Cu-TiO_2	78.16
5%Cu-TiO_2	74.26

Supplementary Table 2 | Detailed information on Ti–O and Ti–Ti contribution from Ti K-edge EXAFS fitting data.

Sample	Scattering Path	CN[a]	R (Å)[b]	σ^2 (Å^2)[c]
0%Cu-TiO_2	Ti–O	3.98 ±0.31	1.93 ±0.02	0.00599
	Ti–Ti	2.59 ±0.44	2.96 ±0.01	0.00421
2%Cu-TiO_2	Ti–O	3.08 ±0.26	1.93 ±0.02	0.00311
	Ti–Ti	2.25 ±0.48	2.97 ±0.01	0.00322
TiO_2 (anatase)	Ti–O	4 (fixed)	1.94 ±0.02	0.00525
	Ti–Ti	2 (fixed)	2.97 ±0.01	0.00418
Ti foil	Ti–Ti	6 (fixed)	2.88 ±0.03	0.00670
	Ti–Ti	6 (fixed)	2.94 ±0.03	0.00670

[a]CN, the coordination numbers. [b]R, the bonding distance. [c]σ^2, the Debye-Waller factor.

Supplementary Table 3 | Detailed information on Cu–O and Cu–Cu/Ti contribution from Cu K-edge EXAFS fitting data.

Sample	Scattering Path	CN[a]	R (Å)[b]	σ^2 (Å^2)[c]
2%Cu-TiO_2	Cu–O	3.82 ±0.22	1.95 ±0.01	0.00674
	Cu–Cu/Ti	1.02 ±0.25	2.80 ±0.01	0.00342
Cu_2O	Cu–O	2 (fixed)	1.84 ±0.01	0.00333
	Cu–Cu	12 (fixed)	3.06 ±0.03	0.02796
CuO	Cu–O	4 (fixed)	1.95 ±0.01	0.00541
Cu foil	Cu–Cu	12 (fixed)	2.54 ±0.01	0.00871

[a]CN, the coordination numbers. [b]R, the bonding distance. [c]σ^2, the Debye-Waller factor.

Supplementary Table 4 | Elemental quantitative analysis for 2%Cu-TiO_2 catalyst before and after reaction using EDS.

Element percentage (atom%)	Before reaction	After 4 cycles of reaction
O	76.20	74.46
Ti	22.77	24.68
Cu	1.03	0.86

Supplementary Table 5 | Performance comparison with the representative work of photocatalytic methane halogenation using alkali halides as halogen source.

Catalysts	Light source	CH_3Cl selectivity	Yield after 4th cycle	Ref.
Ag_2O-loaded La-doped $NaTaO_3$	254 nm UV lamp	56.5%	~12%	[7]
Cu-doped TiO_2	Xe lamp	83.7%	82%	This work

Supplementary Table 6 | Comparison with the representative work of thermocatalytic methane halogenation.

	Catalysts	Halogen source	Reaction temperature	CH_4 conversion	CH_3Cl or CH_3Br selectivity	Ref.
Thermocatalysis	CeO_2	HCl	480 ℃	12%	66%	[8]
Thermocatalysis	$(VO)_2P_2O_7$	HBr	400 ℃	<7%	92%	[9]
Photocatalysis	Cu-TiO_2	NaCl	~30 ℃	2.4%	83.7%	This work

Supplementary Table 7 | The route for conversion of methyl halide into methanol and value-added pharmaceutical intermediate agents.

Entry	Reactant	Reaction conditions	Products	Yield	Applications
1	NaOH	60–80 ℃, ambient	CH_3OH	84%	Fuels & chemicals
2	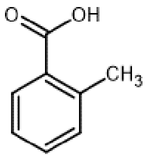	NaOH, DMF, rt, 0.5 h then CH_3Br, 0 ℃–rt, 18 h	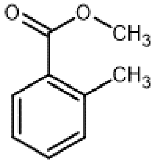	85%	Intermediates for anti-cancer drugs
3	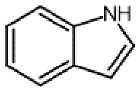	NaOH, DMF, rt, 0.5 h then CH_3Br, 0 ℃–rt, 18 h	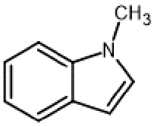	96%	

Supplementary references

1 Colón, G., Maicu, M., Hidalgo, M. C. & Navío, J. A. Cu-doped TiO_2 systems with improved photocatalytic activity. *Appl. Catal. B* **67**, 41-51 (2006).

2 Lee, B.-H. et al. Reversible and cooperative photoactivation of single-atom Cu/TiO_2 photocatalysts. *Nat. Mater.* **18**, 620-626 (2019).

3 Neubert, S. et al. Highly efficient rutile TiO_2 photocatalysts with single Cu(ii) and Fe(iii) surface catalytic sites. *J. Mater. Chem. A* **4**, 3127-3138 (2016).

4 Hur, S. G., Park, D. H., Kim, T. W. & Hwang, S.-J. Evolution of the chemical bonding nature of ferroelectric bismuth titanate upon cation substitution. *Appl. Phys. Lett.* **85**, 4130-4132 (2004).

5 Kim, T. W., Hwang, S.-J., Park, Y., Choi, W. & Choy, J.-H. Chemical bonding character and physicochemical properties of mesoporous zinc oxide-layered titanate nanocomposites. *J. Phys. Chem. C* **111**, 1658-1664 (2007).

6 Silva, L. A., Ryu, S. Y., Choi, J., Choi, W. & Hoffmann, M. R. Photocatalytic hydrogen production with visible light over Pt-interlinked hybrid composites of cubic-phase and hexagonal-phase CdS. *J. Phys. Chem. C* **112**, 12069-12073 (2008).

7 Li, D. et al. Photocatalytic chlorination of methane using alkali chloride solution. *ACS Catal.* **12**, 7004-7013 (2022).

8 He, J. et al. Transformation of methane to propylene: A two-step reaction route catalyzed by modified CeO_2 nanocrystals and zeolites. *Angew. Chem. Int. Ed.* **51**, 2438-2442 (2012).

9 Paunović, V., Zichittella, G., Moser, M., Amrute, A. P. & Pérez-Ramírez, J. Catalyst design for natural-gas upgrading through oxybromination chemistry. *Nat. Chem.* **8**, 803-809 (2016).
